# Novel 2,4-Pyrimidinediamine Derivative as Potent EGFR Inhibitor for NSCLC Treatment

**DOI:** 10.3390/molecules31193440

**Published:** 2026-09-27

**Authors:** Shiqi Jia, Xiaolin Lv, Jia Lv, Yiran Wu

**Affiliations:** 1 University of Science and Technology of China, Hefei 230026, China; 2 Changchun University of Science and Technology College of Optical and Electronic Information, Changchun 130114, China; 18813184194@163.com (X.L.); 13353140675@163.com (J.L.); 3 iHuman Institute, ShanghaiTech University, Shanghai 201210, China; wuyr@shanghaitech.edu.cn

**Keywords:** NSCLC, SPR, H2228, EGFR

## Abstract

Aberrant activation of EGFR drives the progression of non-small-cell lung cancer (NSCLC). As one of the most prevalent driver genes in NSCLC, EGFR represents a critical therapeutic target for patients with NSCLC. In this study, we synthesized 2,4-pyrimidinediamine derivative ZX-3. Compared to its lead compound ceritinib, which has weak cytotoxicity for EGFR-driven NSCLC, ZX-3 exhibited potent cytotoxicity against EGFR-driven NSCLC. We further investigated the antitumor activity and mechanisms of ZX-3 both in vitro and in vivo. Through high-throughput target screening using RTK protein arrays and surface plasmon resonance (SPR) assays, it was demonstrated that ZX-3 reversibly targets the EGFR protein and inhibits its downstream PI3K/AKT signaling pathway. In vitro experiments revealed that ZX-3 suppresses cell colony formation in H2228 cells, induces G1-phase cell cycle arrest, and promotes apoptosis. In in vivo xenograft studies, ZX-3 treatment significantly suppressed tumor growth without obvious toxicity. Collectively, ZX-3 represents a reversible EGFR inhibitor based on borylation modification and possesses favorable antitumor activity both in vitro and in vivo, offering a novel strategy for the subsequent development of EGFR-targeted inhibitors.

## 1. Introduction

Cancer represents a severe disease threatening human life and health. Lung cancer ranks as the most prevalent cancer subtype, accounting for 12.4% of all newly diagnosed malignant cases [1]. Non-small-cell lung cancer (NSCLC) accounts for approximately 85% of all lung cancer cases [2]. Although most patients achieve a prominent initial therapeutic response to surgical cytoreduction and platinum-based combination chemotherapy [3,4], tumor cells gradually develop chemotherapy resistance during treatment. This resistance severely impairs therapeutic efficacy and patient prognosis, ultimately leading to tumor recurrence [5,6].

Epidermal growth factor receptor (EGFR) is one of the predominant driver genes of NSCLC [7]. EGFR is aberrantly overexpressed in numerous cancer subtypes and accelerates tumor progression [8]. Located on the cell membrane surface, EGFR consists of three functional domains [9,10]: an extracellular ligand-binding domain, a transmembrane domain, and an intracellular kinase domain. When binding to a ligand, EGFRs undergo dimerization. Dimerized EGFR subsequently undergoes autophosphorylation at tyrosine residues including Y992, Y1045, Y1068, Y1148 and Y1173, which further activates intracellular kinase signaling cascades such as PI3K/AKT [11,12] and JAK/STAT, and finally facilitates the growth, proliferation and differentiation of tumor cells.

2,4-pyrimidinediamine and 2-aminopyrimidine scaffolds are widely adopted in reported EGFR inhibitors such as **1** (alflutinib) [13], **2** (osimertinib) [14], **3** (mobocertinib) [15], **4** [16] and **5** [17] (Figure 1). Herein, we retained the 2,4-pyrimidinediamine scaffold and introduced a boronate moiety onto ceritinib, which has a 2,4-pyrimidinediamine scaffold. Ceritinib (**6**) is clinically approved as an ALK inhibitor for the treatment of non-small-cell lung cancer and has weak EGFR targeting activity (IC_50_ > 200 nM) [18]. Ceritinib (**6**) has weak therapeutic efficacy against EGFR-driven NSCLC. To improve the therapeutic potency for this type of NSCLC, we performed boronation modification on ceritinib. After boronate modification, the derivative ZX-3 exhibits remarkably enhanced targeting activity toward EGFR. The boron atom of the boronate moiety possesses an empty sp2 orbital [19,20], enabling reversible covalent interactions with proximal nucleophilic residues on target proteins. If nucleophilic residues are distant from the boronate moiety, the oxygen atoms of this moiety may engage in reversible hydrogen-bonding interactions with hydroxyl or amine groups of nucleophilic residues, instead of forming reversible covalent adducts with boron.

Advances in computational biology have enabled researchers to further investigate the molecular mechanisms between target and drug molecules. Accordingly, computational strategies including molecular docking and molecular dynamics simulations are increasingly adopted in relevant studies. As a key tool within computer-aided drug design, molecular docking simulates the three-dimensional binding mode between ligands and receptors to predict their molecular recognition. It permits rapid lead compound screening and quantitative evaluation of binding affinity between small molecules and target proteins, thus providing theoretical support for novel drug discovery and development.

Herein, we investigated boronate-modified 2,4-pyrimidinediamine derivative ZX-3, which exhibited prominent cytotoxicity against NSCLC cells. Mechanistically, ZX-3 targets EGFR, inhibits activation of the downstream PI3K/AKT signaling cascade, suppresses colony formation, induces G1-phase cell cycle arrest, and promotes tumor cell apoptosis. Compared to ceritinib, ZX-3 improves cytotoxicity for EGFR-driven NSCLC. In vivo xenograft studies validate that ZX-3 significantly suppresses tumor growth via inactivating the EGFR/PI3K/AKT signaling pathway.

## 2. Results

### 2.1. Chemistry

Under basic conditions, intermediate **5** undergoes nucleophilic substitution with ceritinib to yield **ZX-3** (Figure 2). Compound **6** underwent a copper-catalyzed coupling reaction to yield **7**, which reacts with FmocCl under basic conditions to protect the amino group of **7** to yield **8**. Subsequently, the nitro group of **8** was reduced to an amino group by zinc dust, providing intermediate **9**. Intermediate **9** then underwent nucleophilic substitution under acidic conditions to yield compound **ZX-3a**. Deprotection of **ZX-3a** under basic conditions removed the protecting group, affording **ZX-3b** (Figure 3).

### 2.2. ZX-3 Cytotoxicity

To achieve boronation modification of the ceritinib scaffold for improved EGFR targeting activity, we first analyzed the binding mode between ceritinib and EGFR. Since no co-crystal structure of the ceritinib–EGFR complex is available to directly illustrate their interaction, docking assays were performed to investigate the interaction between EGFR and ceritinib, ensuring the rational design of boronate modification sites. Docking results revealed that the sulfone moiety of ceritinib forms a hydrogen bond with the Lys704 residue of EGFR, while its piperidine ring forms an additional hydrogen bond with EGFR Asp831 (Figure 4). Furthermore, multiple nucleophilic residues, including Thr766, Lys721, Lys851 and Thr830, are located in proximity to the piperidine ring. These residues are capable of forming reversible covalent bonds or hydrogen bonds with boronate ester moieties.

ZX-3 was modified in the piperidine ring of ceritinib, which may be beneficial to the interaction between ZX-3 and EGFR, achieving improved cytotoxic potency. Based on this strategy, we investigated ZX-3 cytotoxicity (Table 1). ZX-3 exhibited significantly improved cytotoxicity compared with ceritinib in the H2228 and MCF7 cell lines (Figure 5A). ZX-3 also exhibited enhanced cytotoxicity at both 48 h and 72 h after treatment (Figure 5B). In the H2228 cell line, ceritinib exhibited cytotoxicity with an IC_50_ of 1.18 ± 0.14 μM, and EGFR receptor inhibitor gefitinib exhibited cytotoxicity with an IC_50_ of 2.46 ± 0.33 μM, while ZX-3 exhibited enhanced potency with an IC_50_ of 0.65 ± 0.11 μM. A similar trend was also observed in MCF7 cells; compared with ceritinib (IC_50_ = 4.33 ± 0.16 μM) and gefitinib (IC_50_ = 2.65 ± 0.25 μM), ZX-3 (IC_50_ = 1.26 ± 0.12 μM) cytotoxicity was increased. Furthermore, compared with ceritinib (IC_50_ = 2.54 ± 0.21 μM), ZX-3 (Table 2) exhibited reduced cytotoxicity toward human normal liver cell line LO2 (IC_50_ = 4.28 ± 0.19 μM).

For ZX-3b (Table 1), it retains the 2,4-pyrimidinediamine scaffold, without the piperidine ring and boronate ester modification, and exhibits reduced cytotoxic potency compared with ceritinib. Its IC_50_ value was 6.25 ± 0.28 μM in H2228 cells and 8.36 ± 0.51 μM in MCF7 cells. ZX-3a maintains the 2,4-pyrimidinediamine scaffold while bearing a bulky Fmoc group, leading to a remarkable decline in cytotoxic potency. The IC_50_ values of ZX-3a exceeded 64 μM in both the H2228 and MCF7 cell lines. For the EGFR kinase inhibition assay (Table 3), ZX-3a, ZX-3b, and ceritinib do not show significant inhibition activity (IC_50_ > 200 nM). ZX-3 (Figure 5C) shows increased inhibition activity (IC_50_ = 12.36 ± 0.69 nM), compared to selective EGFR receptor inhibitor gefitinib (IC_50_ = 36.12 ± 1.35 nM). Although ZX-3a, ZX-3b, and ceritinib have 2,4-pyrimidinediamine scaffolds, increased inhibition activity for EGFR cannot be observed. This means that the 2,4-pyrimidinediamine scaffold may serve as a conserved skeleton for molecular anchoring, while the improved cytotoxic potency of ZX-3 may be attributed to the introduced boronate moiety.

### 2.3. ZX-3 Targets EGFR

To investigate the target of ZX-3, first, we performed high-throughput target screening using an RTK phosphorylation antibody array, which enables simultaneous quantification of phosphorylation levels across 71 RTK family proteins [21]. We focused on RTK family proteins with decreased phosphorylation levels following ZX-3 treatment in H2228. Although H2228 is generally regarded as an EML4-ALK fusion mutant cell line, data from The Human Protein Atlas and UniProt databases indicate that receptor tyrosine kinase (RTK) family proteins including EGFR [22,23], ACK1, IGF1R and ROS1 are also abundantly expressed in H2228 cells. Notably, the phosphorylation levels of EGFR were significantly reduced, while only two protein phosphorylation levels showed increased levels. Other RTK family proteins expressed in the H2228 cell line, such as ALK, IGF1R, and ROS1, exhibited no significant changes in phosphorylation levels compared to the control group, preliminarily indicating that ZX-3 targets EGFR (Figure 6A,B).

Subsequently, surface plasmon resonance (SPR) experiments were conducted to investigate the binding affinity and interaction mechanism between ZX-3 and EGFR (Figure 6D). The results revealed a binding rate constant (k_a_ [M^−1^·s^−1^]) of 1.24 × 10^7^, a dissociation rate constant (k_d_ [s^−1^]) of 0.13, and an equilibrium dissociation constant (K_D_) of 10.63 nM. These findings indicate strong binding affinity between ZX-3 and EGFR. ZX-3 rapidly binds to EGFR during the binding phase, and completely dissociates from EGFR during the dissociation phase, demonstrating preliminarily that the interaction is reversible [24]. The reversible binding mode will be further validated by NMR or Hydrogen/Deuterium exchange mass spectrometry (HDX-MS) in our future work. In contrast, irreversible covalent binding would require slow formation of covalent bonds between the drug molecule and the target site, resulting in sluggish binding during the binding phase. During dissociation, the stable covalent bond makes complete dissociation unlikely within a short time. Thus, it is confirmed that ZX-3 exhibits robust and reversible binding with EGFR.

Western blot analysis further confirmed that ZX-3 targets EGFR and thereby inhibits its phosphorylation (Figure 6C). At a low concentration of 0.5 μM, EGFR phosphorylation levels were significantly reduced compared to the control, phosphorylation was almost completely inhibited, and then AKT phosphorylation was decreased. As the concentration increased, at 1 μM, both EGFR and its downstream pathway protein AKT exhibited complete inhibition of phosphorylation. Molecular docking simulations demonstrated that ZX-3 readily binds to the kinase pocket of EGFR (Figure 6E), where the oxygen atom of its borate group forms a hydrogen bond with the Thr830 residue in the EGFR kinase domain, enhancing ZX-3 binding affinity to EGFR. The docking score for ZX-3 bound to EGFR is −9.3 kcal/mol, that of ceritinib bound to EGFR is −7.2 kcal/mol, and that of gefitinib bound to EGFR is −8.5 kcal/mol (Table 4). The result of the docking assay is consistent with the result of the kinase inhibition assay. ZX-3 exhibited enhanced target-binding activity compared with ceritinib and gefitinib in the docking assay.

### 2.4. Effects of ZX-3 on the Proliferation of Tumor Cells

It can be observed that ZX-3 induces apoptosis in a dose-dependent manner. Compared to the control group, the low-concentration ZX-3 group (0.5 μM) exhibited a total apoptosis rate of 16.66%, comprising 10.00% early apoptotic cells and 6.66% late apoptotic cells; at high concentration (2 μM), the total apoptosis rate increased to 62.02%, with early apoptotic cells accounting for 22.12% and late apoptotic cells for 39.90% (Figure 7). Meanwhile, the ceritinib [25] group at a treatment concentration of 0.5 μM exhibited a total apoptosis of 17.73%, comprising 3.74% early apoptotic cells and 13.99% late apoptotic cells. For the ceritinib group at a treatment concentration of 2 μM, the total apoptosis rate increased to 33.29%, with early apoptotic cells accounting for 3.38% and late apoptotic cells for 29.91%. Additionally, ZX-3 has significant cytotoxicity against high-EGFR-expressing cell lines, including EGFR-driven NSCLC: the IC_50_ values were 0.65 ± 0.11 μM for H2228 cells, 0.84 ± 0.21 μM for A549 cells, and 0.68 ± 0.07 μM for HeLa cells; for the low-EGFR-expression cell line [26], H520 had IC_50_ values of 5.22 ± 0.21 μM, indicating its potent cytotoxic effect on EGFR-high tumor cell lines due to its targeted action on EGFR and decreased cytotoxicity for the low-EGFR-expression cell line (Figure 8C).

In H2228 cells, ZX-3 suppressed colony formation in a dose-dependent manner (Figure 8D). In the low-concentration ZX-3 group (0.5 μM), the colony formation inhibitory rate was 64.92%. When the treatment concentration increased to 2 μM, the inhibition rate of colony formation was 94.76%, while the colony formation inhibitory rate of the ceritinib group at 0.5 μM was 26.35% and reached 72.61% at 2 μM. Finally, ZX-3 arrested the cell cycle in the G1 phase in a dose-dependent manner (Figure 8A). At the low concentration (0.5 μM), the proportion of cells arrested at the G1 phase was 35.09%. At the high concentration (2 μM), the proportion of cells arrested at the G1 phase was 43.78%.

### 2.5. In Vivo Antitumor Activity of ZX-3

We further evaluated the in vivo antitumor efficacy of ZX-3 using the H2228 xenograft model (Figure 9). Mice were treated with ZX-3 via continuous administration for 14 consecutive days. Tumor growth was suppressed in the low-dose ZX-3 group (15 mg/kg), with a tumor growth inhibition (TGI) rate of 66.59%. Notably, the high-dose group (30 mg/kg) exhibited remarkable tumor growth suppression, achieving a TGI value of 85.48%. No obvious body weight loss was observed throughout the treatment period, suggesting the absence of obvious acute systemic toxicity. Consistent with in vitro assays, Western blot assays further revealed significant reductions in phosphorylated p-EGFR and p-AKT levels in tumor tissues following ZX-3 administration.

## 3. Discussion

2,4-pyrimidinediamine scaffolds are widely adopted in reported EGFR inhibitors such as almonertinib, osimertinib, furmonertinib and mobocertinib. This scaffold can bind into the ATP binding pocket within the kinase domain of epidermal growth factor receptor (EGFR) via multiple hydrogen bonds and hydrophobic interactions, acting as the critical structural motif responsible for EGFR inhibitory potency. In the present work, we investigate the cytotoxicity and mechanism of action of ZX-3; ceritinib, a clinically available ALK inhibitor, was selected as the lead compound. Ceritinib intrinsically bears the 2,4-pyrimidinediamine scaffold yet exhibits extremely weak EGFR-suppressive activity (IC_50_ > 200 nM), which restricts its therapeutic application in EGFR-driven non-small-cell lung cancer (NSCLC).

Ceritinib derivative ZX-3 modified with a boronate moiety displays markedly enhanced EGFR targeting potency. Molecular docking verifies that ZX-3 forms hydrogen bonds with the nucleophilic residue Thr830 located in the EGFR binding pocket, thereby strengthening its binding affinity toward EGFR protein. ZX-3 exerts more effective cytotoxicity in EGFR-driven NSCLC compared with ceritinib. Nevertheless, the current study only evaluated the antiproliferative activity of ZX-3 in EGFR wild-type cell lines. The efficacy of ZX-3 against NSCLC cell lines with EGFR mutations (EGFR^L858R^, EGFR^T790M^ and EGFR^C797S^) remains uncharacterized. Furthermore, we have not investigated whether ZX-3, as a reversible inhibitor, could overcome drug resistance induced by mutations at the covalent binding site within the EGFR kinase domain that renders covalent EGFR inhibitors resistant. Future research will systematically explore the cellular inhibitory activity of ZX-3 against a panel of EGFR mutant variants.

## 4. Materials and Methods

All reagents and solvents were from Bidepharm (Shanghai, China). ^1^H NMR spectra were collected at 400 MHz by a Bruker AVANCE NEO 400 spectrometer (Bruker, Billerica, MA, USA), while ^13^C NMR spectra were acquired at 101 MHz using the same instrument. ^11^B NMR measurements were conducted on a JEOL JNM-ECZL400S spectrometer at 128 MHz (JEOL Ltd., Tokyo, Japan). Chemical shifts (δ) for ^1^H, ^13^C and ^11^B NMR are reported in ppm, referenced to residual solvent peaks of CDCl_3_ (δ 7.26 for ^1^H; δ 77.16 for ^13^C). All coupling constants (J values) are reported in Hertz (Hz). The following abbreviations represent the multiplicity: s, singlet; d, doublet; t, triplet; q, quartet; and m, multiplet. High-resolution mass spectrometry (HRMS) data were acquired with a Thermo Scientific Q Exactive mass spectrometer under positive electrospray ionization mode. The spectrograms can been seen in Supplementary Materials. All final compounds used for biological evaluation were of >95% purity, as determined by high-performance liquid chromatography (HPLC). 

### 4.1. Synthesis

(E)-3-(4,4,5,5-tetramethyl-1,3,2-dioxaborolan-2-yl) allyl-4-(4-((5-chloro-4-((2-(isopropylsulfonyl) phenyl) amino) pyrimidin-2-yl) amino)-5-isopropoxy-2-methylphenyl) pip-eridine-1-carboxylate (**ZX-3**). **7** (31 mg, 0.09 mmol) was added to a solution of ceritinib (50 mg, 0.09 mmol) and DIPEA (*N*,*N*-Diisopropylethylamine) (16 μL, 0.18 mmol) in DCM (Dichloromethane) at 0 °C. The reaction solution was stirred at room temperature for 4 h. After completion of the reaction, the mixture was quenched with water, and extracted with DCM. The combined organic layers were washed with saturated brine, dried over anhydrous Na_2_SO_4_, and filtered. The solvent was concentrated under reduced pressure. The crude residue was purified via silica gel column chromatography (MeOH (Methanol):DCM = 1:50) to yield ZX-3 (37 mg, 54.2%). ^1^H NMR (400 MHz, CDCl_3_) δ 9.50 (s, 1H), 8.58 (dd, J = 8.5, 1.0 Hz, 1H), 8.16 (s, 1H), 8.02 (s, 1H), 7.93 (dd, J = 7.9, 1.6 Hz, 1H), 7.68–7.52 (m, 2H), 7.26 (t, 1H), 6.79–6.62 (m, 2H), 5.70 (dt, J = 18.2, 1.9 Hz, 1H), 4.72 (dd, J = 4.4, 1.9 Hz, 2H), 4.57 (hept, J = 6.0 Hz, 1H), 4.35 (d, J = 13.2 Hz, 2H), 3.26 (hept, J = 6.9 Hz, 1H), 2.96–2.76 (m, 3H), 2.17 (s, 3H), 1.77–1.65 (m, 2H), 1.59–1.43 (m, 2H), 1.37 (d, J = 6.1 Hz, 6H), 1.32 (d, J = 6.9 Hz, 6H), 1.28 (s, 12H). ^13^C NMR (101 MHz, CDCl_3_) δ 157.5, 155.4, 155.0, 147.2, 144.8, 138.5, 137.0, 134.7, 131.3, 127.7, 126.8, 125.0, 123.7, 123.2, 120.7, 110.8, 105.8, 83.4, 71.6, 66.3, 55.5, 44.9, 38.1, 32.6, 29.7, 24.9, 24.8, 22.3, 19.0, 15.4. ^11^B NMR (128 MHz, CDCl_3_) δ 29.26. HRMS (ESI): *m*/*z* calc. for C_38_H_52_BClN_5_O_7_S [M + H]^+^: 768.3363, found 768.3360; *m*/*z* calc. for C_38_H_51_BClN_5_O_7_SNa [M + Na]^+^: 790.3183, found 790.3176; *m*/*z* calc. for C_38_H_51_BClN_5_O_7_SK [M + K]^+^: 806.2932, found 806.2953.

(9H-fluoren-9-yl)methyl (4-((5-chloro-4-((2-(isopropylsulfonyl)phenyl)amino)pyrimidin-2-yl) amino)-5-isopropoxy-2-methylphenyl)carbamate (**ZX-3a**). To a solution of 2,5-dichloro-*N*-(2-(isopropylsulfonyl)phenyl)pyrimidin-4-amine (381 mg, 1.10 mmol) and **11** (402 mg, 1.00 mmol) in isopropanol at 0 °C was added HCl (1N). The reaction mixture was reflux-heated at 80 °C for 12 h. Upon completion, the reaction was quenched with water and extracted with DCM (20 mL × 3). The combined organic layers were washed with saturated brine, dried over anhydrous Na_2_SO_4_, and filtered. The crude residue was purified by silica gel column chromatography (EtOAc (ethyl acetate):PE (Petroleum ether) = 1:3) to afford compound **ZX-3a** (311 mg, 43.8%). ^1^H NMR (500 MHz, CDCl_3_) δ 9.51 (s, 1H), 8.55 (d, J = 8.4 Hz, 1H), 8.16 (s, 1H), 8.05 (s, 1H), 7.93 (d, J = 8.0 Hz, 1H), 7.78 (m, 2H), 7.53–7.67 (m, 4H), 7.42 (m, 2H), 7.33 (m, 2H), 7.28 (m, 1H), 7.25 (m, 1H), 6.35 (s, 1H), 4.51 (m, 2H), 4.29 (m, 1H), 3.26 (hept, J = 6.9 Hz, 1H), 2.09 (s, 3H), 1.38 (d, J = 6.1 Hz, 6H), 1.32 (d, J = 6.9 Hz, 6H). ^13^C NMR (101 MHz, CDCl_3_) δ 157.30, 155.35, 144.85, 143.74, 141.36, 138.39, 134.59, 131.29, 127.79, 127.08, 125.01, 123.71, 123.24, 120.43, 120.07, 105.88, 71.63, 66.97, 55.49, 47.19, 22.14, 17.32, 15.37. HRMS (ESI): *m*/*z* calc. for C_38_H_38_ClN_5_O_5_SNa [M + Na]^+^: 734.2174, found 734.2182.

N^2^-(4-amino-2-isopropoxy-5-methylphenyl)-5-chloro-N^4^-(2-(isopropylsulfonyl)phenyl)pyrimidine-2,4-diamine (**ZX-3b**). NEt_3_ (305 μL, 2.2mmol) was added in a solution of **ZX-3a** (311 mg, 0.44 mol) in DCM at 0 °C. The reaction mixture was stirred at room temperature for 24 h. Upon completion, the reaction was quenched with water and extracted with DCM. The combined organic layers were washed with brine, dried over anhydrous Na_2_SO_4_ and filtered. The crude residue was purified by silica gel column chromatography (EtOAc:PE = 1:1) to afford compound **ZX-3b** (196 mg, 91.5%). ^1^H NMR (500 MHz, CDCl_3_) δ 9.50 (s, 1H), 8.60 (d, J = 8.4 Hz, 1H), 8.11 (s, 1H), 7.91 (dd, J = 8.0, 1.6 Hz, 1H), 7.77 (s, 1H), 7.58 (dd, J = 8.7, 1.7 Hz, 1H), 7.30–7.23 (m, 2H), 6.32 (s, 1H), 4.47 (m, 1H), 3.26 (m, 1H), 2.03 (s, 3H), 1.34 (d, J = 6.1 Hz, 5H), 1.31 (d, J = 6.9 Hz, 6H). ^13^C NMR (101 MHz, CDCl_3_) δ 157.90, 155.33, 155.23, 146.44, 139.90, 138.65, 134.63, 131.21, 124.63, 123.63, 122.93, 122.69, 121.34, 114.03, 105.06, 71.58, 55.47, 53.46, 22.28, 17.04, 15.40. HRMS (ESI): *m*/*z* calc. for C_23_H_28_ClN_5_O_3_SNa [M + Na]^+^: 512.1493, found 512.1485; *m*/*z* calc. for C_23_H_29_ClN_5_O_3_S [M + H]^+^: 490.1674, found 490.1671.

### 4.2. Cell Viability Assay

NCI-H2228, MCF7, HeLa, NCI-H520 and A549 cell lines were purchased from Procell. Cells were cultivated in DMEM medium with 10% fetal bovine serum in a 37 °C humidified incubator with 5% CO_2_. The cell line used in this study was authenticated and confirmed to be negative for mycoplasma.

Cell viability was determined by the MTT assay. Briefly, 5000 cells per well were seeded in 96-well plates overnight. Cells were subsequently treated with different concentrations of ZX-3 for 48 h. Then 10 μL of MTT (5 mg/mL) was added to each well, and plates were incubated at 37 °C for 4 h. The medium was then removed, and 150 μL DMSO was added to dissolve precipitates. The optical density (OD) was measured at 490 nm by a SpectraMax i3x microplate reader (Molecular Devices, LLC, San Jose, CA, USA). All statistical analyses were conducted by GraphPad Prism 10.1.2 software.

### 4.3. Cloning Formation Assay

Briefly, 3000 cells per well were seeded in 6-well plates and cultivated overnight. Cells were treated with ZX-3 at concentrations of 0.5, 1, and 2 μM, and culture medium was refreshed every 3 days. After 12 days of colony growth, cells were fixed and stained with crystal violet. Stained colonies were imaged, and the total colony area was quantified using ImageJ1 software.

### 4.4. Cell Apoptosis Assay

First, 2 × 10^5^ H2228 cells per well were seeded into a 6-well plate, and then treated with different concentrations of ZX-3 for 24 h. Apoptosis was detected by Annexin V-FITC Apoptosis Detection Kit I (BestBio, Beijing, China) according to the manufacturer’s instructions. Flow cytometry was used to detect samples and analyze data.

### 4.5. Cell Cycle Assay

First, 2 × 10^5^ H2228 cells per well were seeded into a 6-well plate and treated with different concentrations of ZX-3 for 24 h. Cells were collected, washed twice with ice-cold PBS, and fixed with 70% ethanol at 4 °C overnight. Finally, cells were stained with RNase A and PI (BestBio) following the kit’s instructions, and subsequently analyzed by flow cytometry.

### 4.6. Receptor Tyrosine Kinase Phosphorylation Array

The human receptor tyrosine kinase (RTK) phosphorylation antibody array (RayBiotech, G1, Peachtree Corners, GA, USA) was performed following the manufacturer’s instructions. H2228 cells were treated with ZX-3 (2 μM) for 24 h.

### 4.7. Western Blot Analysis of Cancer Cells

H2228 cells were treated with ZX-3 (0.5, 1, 2 μM) for 24 h, and then collected and lysed by RIPA buffer. Total protein concentrations were determined by a BCA kit following the manufacturer’s protocol. Lysates were combined with loading buffer and boiled at 95 °C for 7 min. Proteins were separated by SDS-PAGE and transferred to PVDF membranes. After blocking in 5% non-fat milk, membranes were incubated with primary antibodies overnight at 4 °C, and then incubated with secondary antibodies for 1 h at room temperature. The following proteins were detected with an ECL reagent: EGFR, p-AKT, AKT (Immunoway, Suzhou, Jiangsu, China), p-EGFR (HUABIO, Hangzhou, Zhejiang, China), and β-actin (Proteintech, Wuhan, Hubei, China).

### 4.8. Surface Plasmon Resonance (SPR)

SPR measurements were performed at 25 °C using a Biacore T200 system (Cytiva, Marlborough, MA, USA). Recombinant EGFR protein [27,28] (E10-112G, SinoBiological, Beijing, China) was covalently immobilized onto a CM5 chip. Serially diluted ZX-3 samples, prepared in HBS-EP+ buffer containing 0.01% DMSO, were injected over the chip surface to acquire binding response signals. The equilibrium dissociation constant (K_D_) was calculated using Biacore Insight Evaluation Software.

### 4.9. Kinase Inhibition Assay

The experiment was conducted in kinase buffers, including 20 mM HEPES pH 7.5, 0.005% Brij35,1 mM DTT, 5 mM MgCl_2_, and 100 mM NaCl. EGFR (Sigma-Aldrich, Saint Louis, MO, USA), substrate polyE4Y1 (Sigma-Aldrich), ATP and compounds were added to 384 plates and incubated at room temperature for 60 min. In the dark, an ADP-Glo reagent (Promega, Tokyo, Japan) was added and incubated at room temperature. Finally, a kinase detection reagent (Promega) was added and incubated at room temperature for 30 min. The plates were read on BioTek (Winooski, VT, USA), and the luminescence was recorded. The IC_50_ value was calculated by GraphPad Prism 4.10.

### 4.10. Docking Analysis

The docking analysis was performed according to a previously reported protocol by Yi Zhou et al. [29]. The crystal structure of EGFR (PDB: 4HJO) was selected to investigate the binding interaction between ZX-3 and EGFR. Molecular docking was performed in Maestro. The EGFR structure was prepared using the Protein Preparation Wizard, including water removal, hydrogenation, and energy minimizations. LigPrep was used to generate stable conformations of ZX-3. Receptor Grid Generation generated the binding pocket of EGFR. Then, ligand docking was performed to predict binding modes between protein and ligand. Docking results were visualized with PyMOLWin.

### 4.11. Tumor Xenograft

All animal experiments were performed under protocols reviewed and approved by the Institutional Animal Care and Use Committee (IACUC) of Jilin University. Male BALB/c nude mice (6–8 week) were obtained from Charles River. H2228 cells [30,31] (8 × 10^6^ cells per mouse) were subcutaneously injected into the right flank of mice. When tumor volumes reached approximately 80 mm^3^, mice were divided into three groups: vehicle control, ZX-3 (15 mg/kg), and ZX-3 (30 mg/kg). ZX-3 was formulated in 0.5% methylcellulose (MC) and administered via oral gavage once daily for 14 consecutive days. Body weight and tumor volume were monitored every two days. Tumor volume was calculated using the following formula: (Length × Width^2^)/2. Mice were sacrificed on day 14, and tumor tissues were excised and weighed.

### 4.12. Statistical Analysis

Results are expressed as mean ± SEM. GraphPad Prism software was used for statistical calculations. One-way ANOVA or Student’s *t*-test was adopted to assess intergroup differences. Statistical significance was defined as *p* < 0.05 [32,33].

## 5. Conclusions

ZX-3 exhibited potent cytotoxicity against non-small-cell lung cancer (NSCLC). We further investigated underlying molecular mechanisms and antitumor activity of ZX-3. It exhibits potent binding affinity toward EGFR with a K_D_ of 10.63 nM. It effectively suppresses EGFR autophosphorylation and markedly reduces the phosphorylation level of AKT, a key downstream protein of the EGFR signaling cascade. In vitro assays demonstrate that ZX-3 inhibits colony formation and induces tumor cell apoptosis and cell cycle arrest in the G1 phase. In vivo xenograft experiments reveal that ZX-3 significantly inhibits tumor growth; the high-dose treatment group (30 mg/kg) achieves a tumor growth inhibition (TGI) rate of 85.48%. Collectively, ZX-3 functions as an effective reversible EGFR inhibitor with prominent antitumor potency and inactivates the PI3K/AKT signaling pathway both in vitro and in vivo, offering a promising strategy for the treatment of NSCLC.

## Figures and Tables

**Figure 1:**
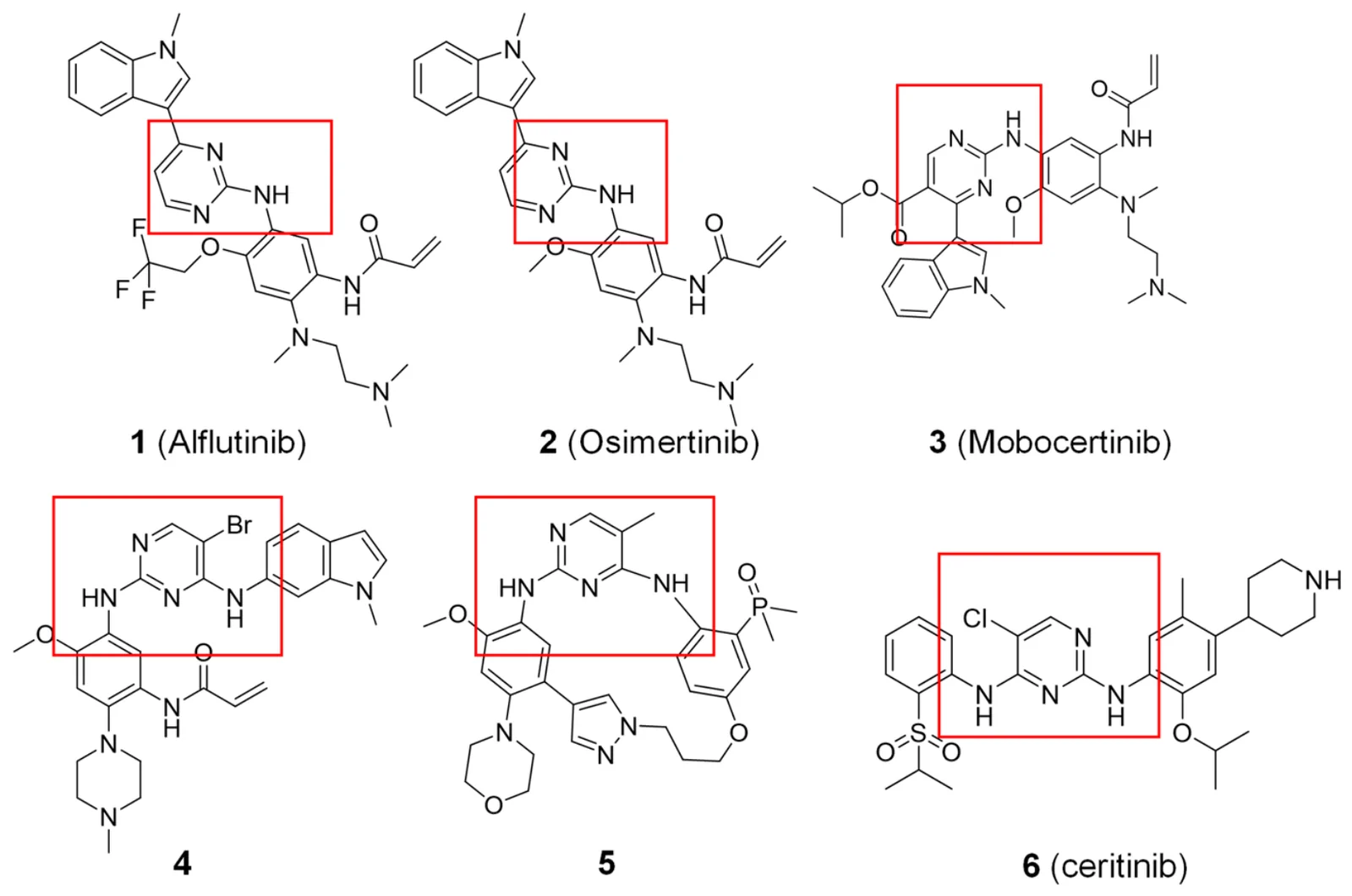
EGFR inhibitors with a 2,4-pyrimidinediamine scaffold.

**Figure 2:**
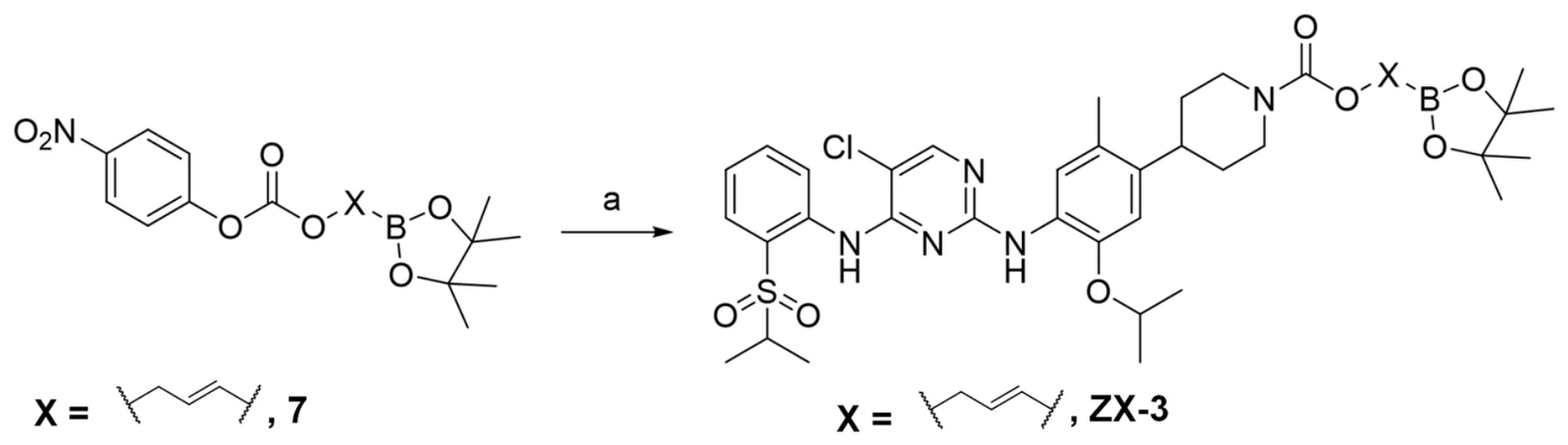
The synthesis of ZX-3. Reagents and conditions: (a) DIPEA, ceritinib, DCM, rt, 6 h.

**Figure 3:**
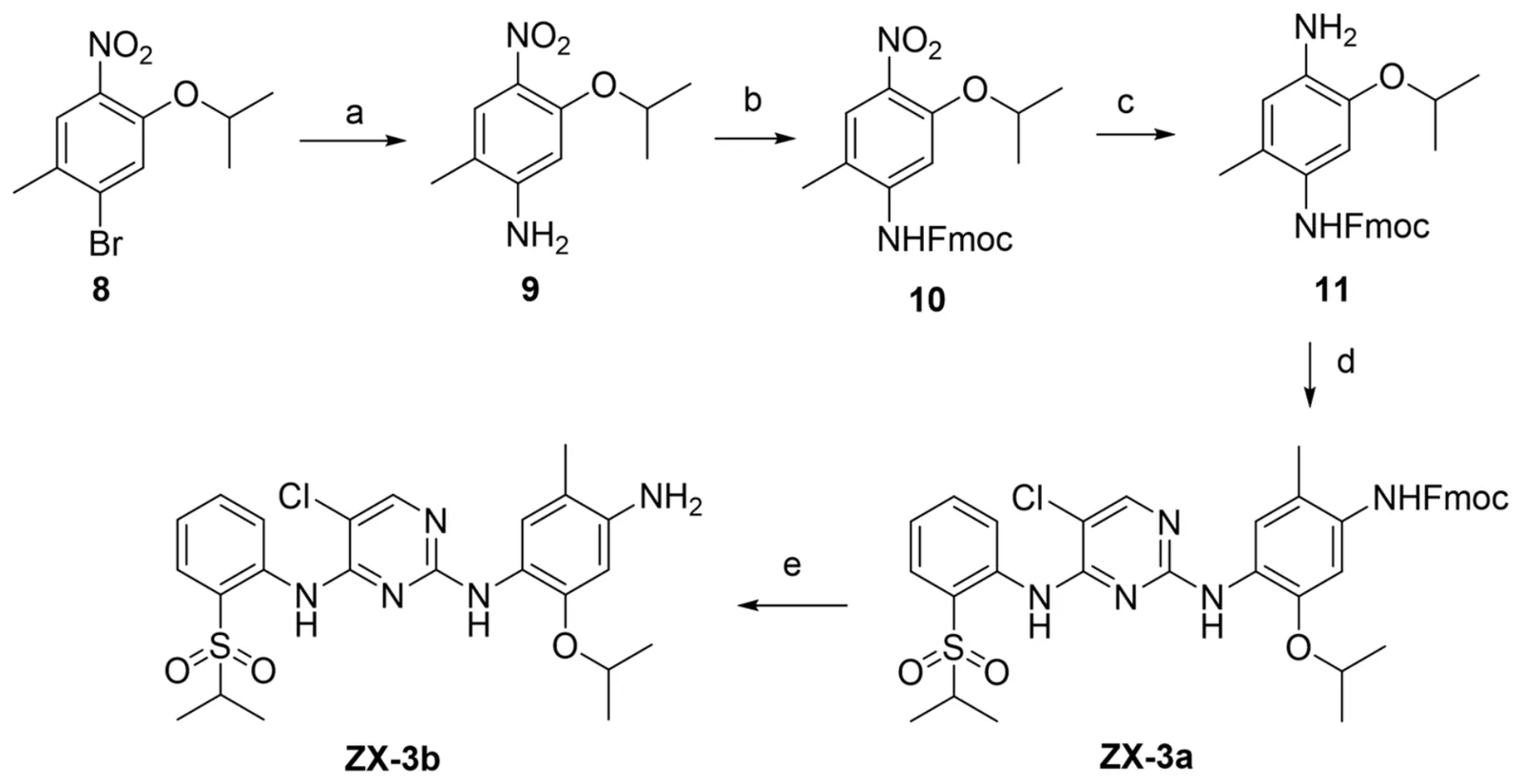
The synthesis of ZX-3a and ZX-3b. Reagents and conditions: (a) L-proline, CuI, NH_3_.H_2_O, K_2_CO_3_, DMSO, 55 °C, N_2_. (b) NaHCO_3_, FmocCl, dioxane, rt, 24 h. (c) Zinc powder, CH_3_COOH, THF, rt, 24 h. (d) HCl (1N), 2,5-dichloro-*N*-(2-(isopropylsulfonyl)phenyl)pyrimidin-4-amine, isopropanol, 80 °C, 12 h. (e) NEt_3_, DCM, rt, 24 h.

**Figure 4:**
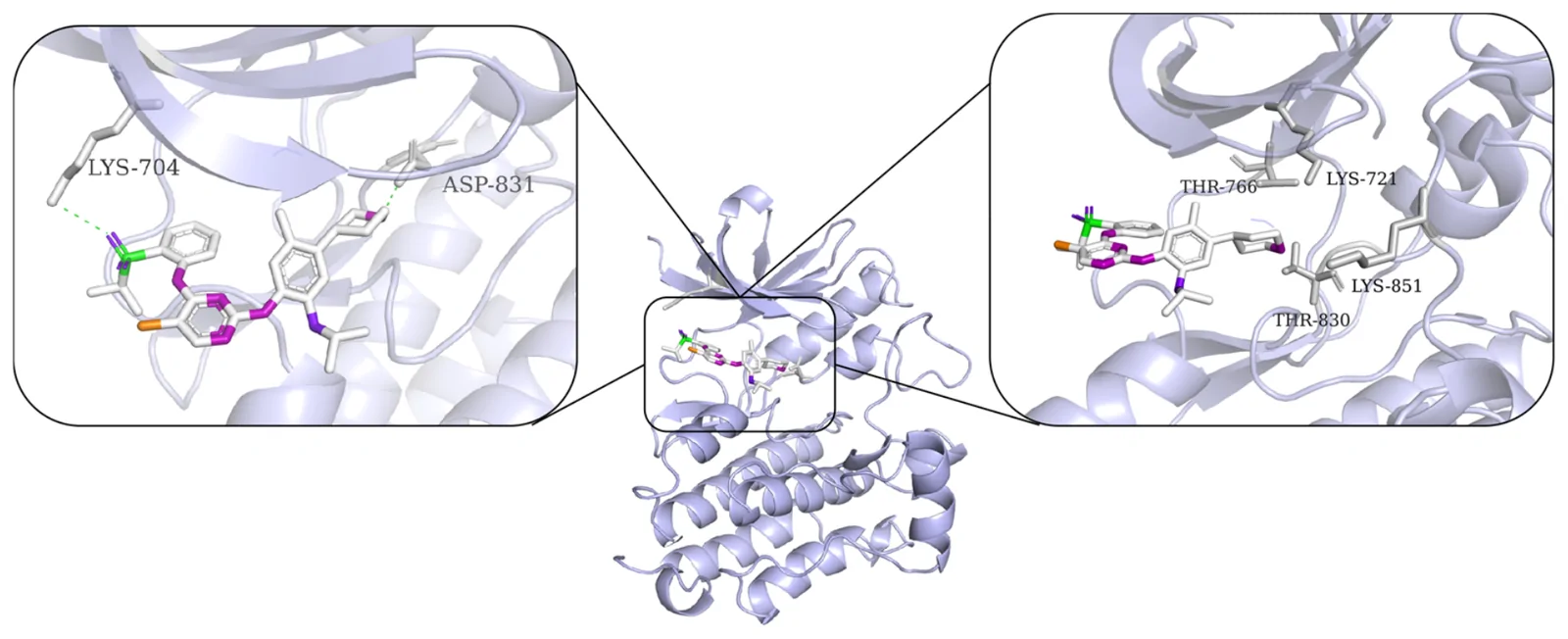
**Left**: the docking of ceritinib and EGFR (PDB: 4HJO). **Right**: The same docking of ceritinib and EGFR (PDB: 4HJO) was rotated 90° counter-clockwise. Green: sulfur atom, blue: oxygen atom, purple: nitrogen atom, orange: chlorine atom.

**Figure 5:**
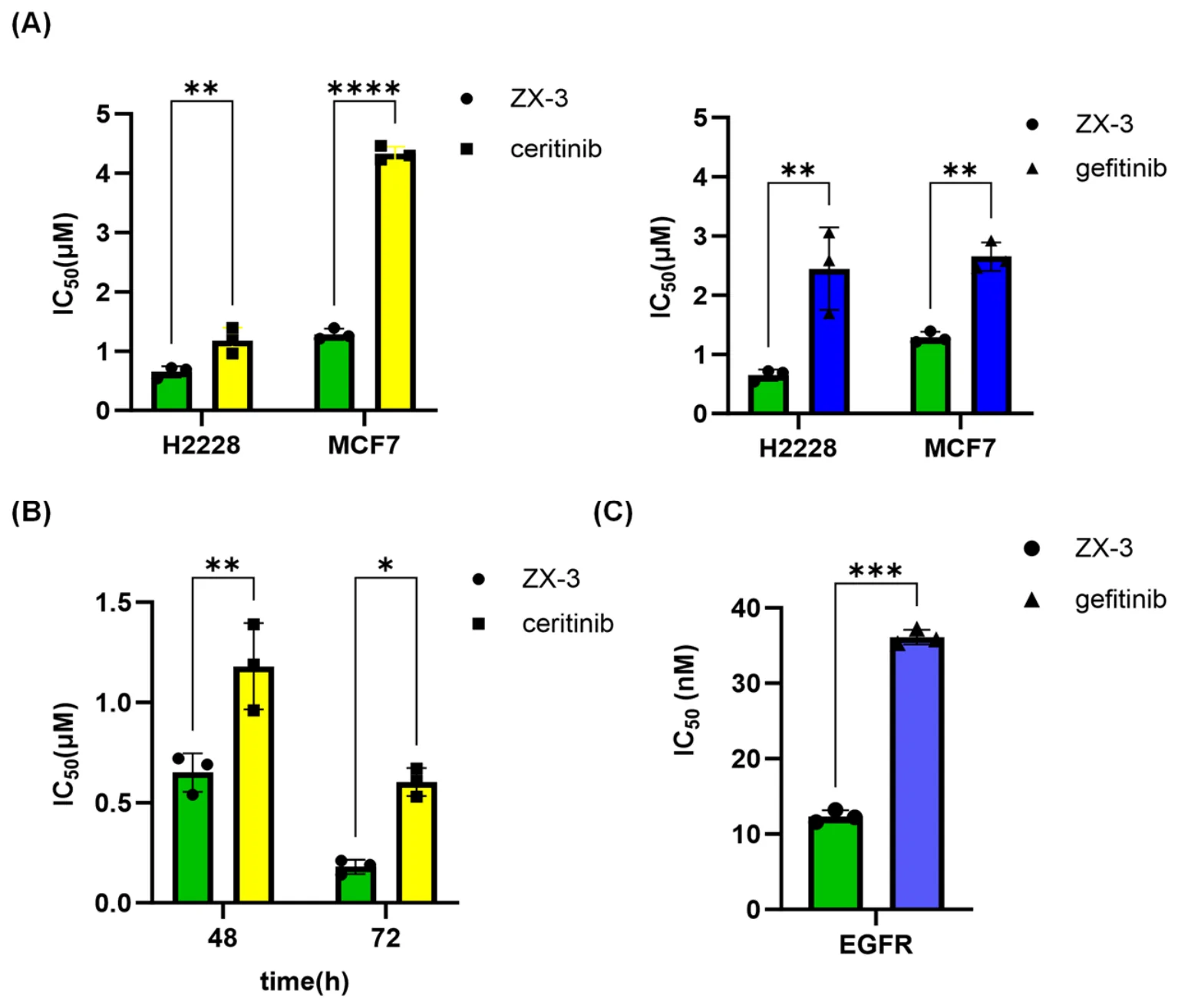
(**A**) Statistical analysis of cytotoxicity in H2228 and MCF7 cell lines after 48 h treatment. (**B**) Statistical analysis of cytotoxicity in H2228 cell line after 48 h and 72 h treatment. (**C**) Statistical analysis of kinase inhibition. Data are expressed as mean ± standard error, *n* ≥ 3. (*), *p* < 0.05; (**), *p* < 0.01; (***), *p* < 0.001; (****), *p* < 0.0001.

**Figure 6:**
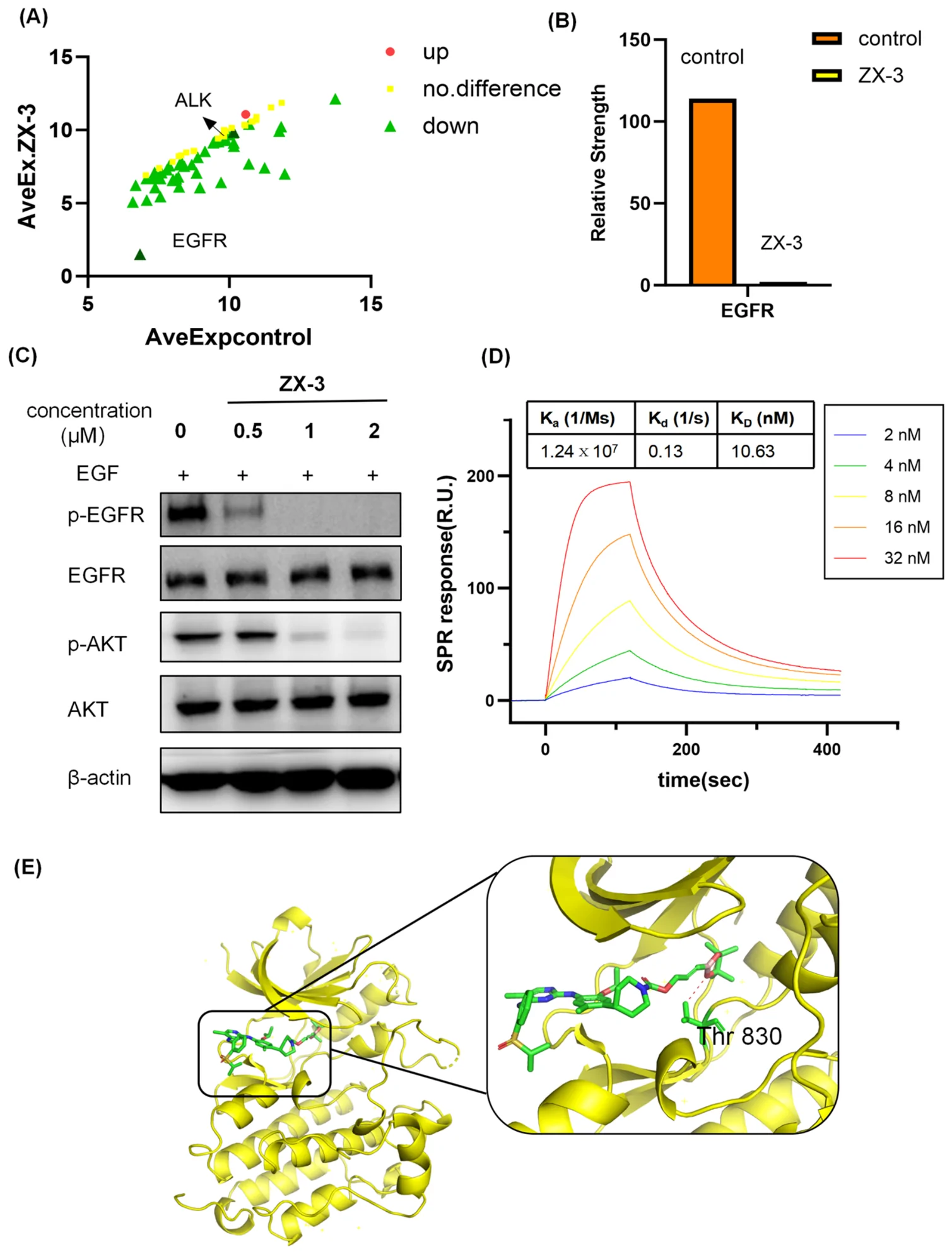
(**A**) Inhibition of phosphorylation in 71 RTK family proteins after treatment with ZX-3 (2 μM). (**B**) EGFR phosphorylation levels following ZX-3 treatment (2 μM). (**C**) Protein blot analysis results at different ZX-3 concentrations. (**D**) EGFR–ZX-3 binding and dissociation determined by surface plasmon resonance (SPR). (**E**) Molecular docking of ZX-3 and EGFR interaction (PDB: 4HJO), yellow: sulfur atom, red: oxygen atom, blue: nitrogen atom, pink: boron atom.

**Figure 7:**
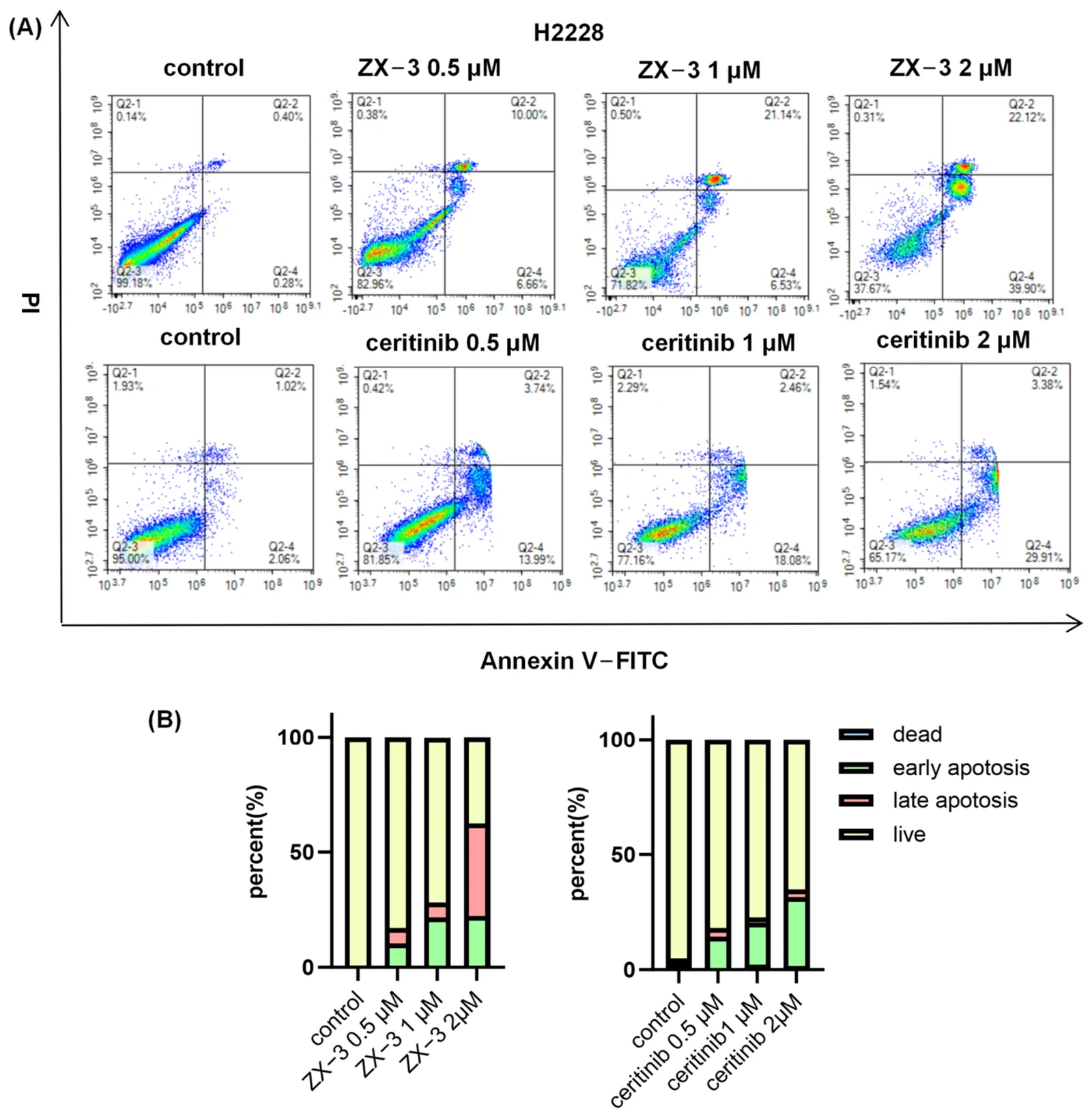
(**A**) ZX-3 and ceritinib induce cell apoptosis after 24 h treatment. (**B**) Quantitative analysis of (**A**).

**Figure 8:**
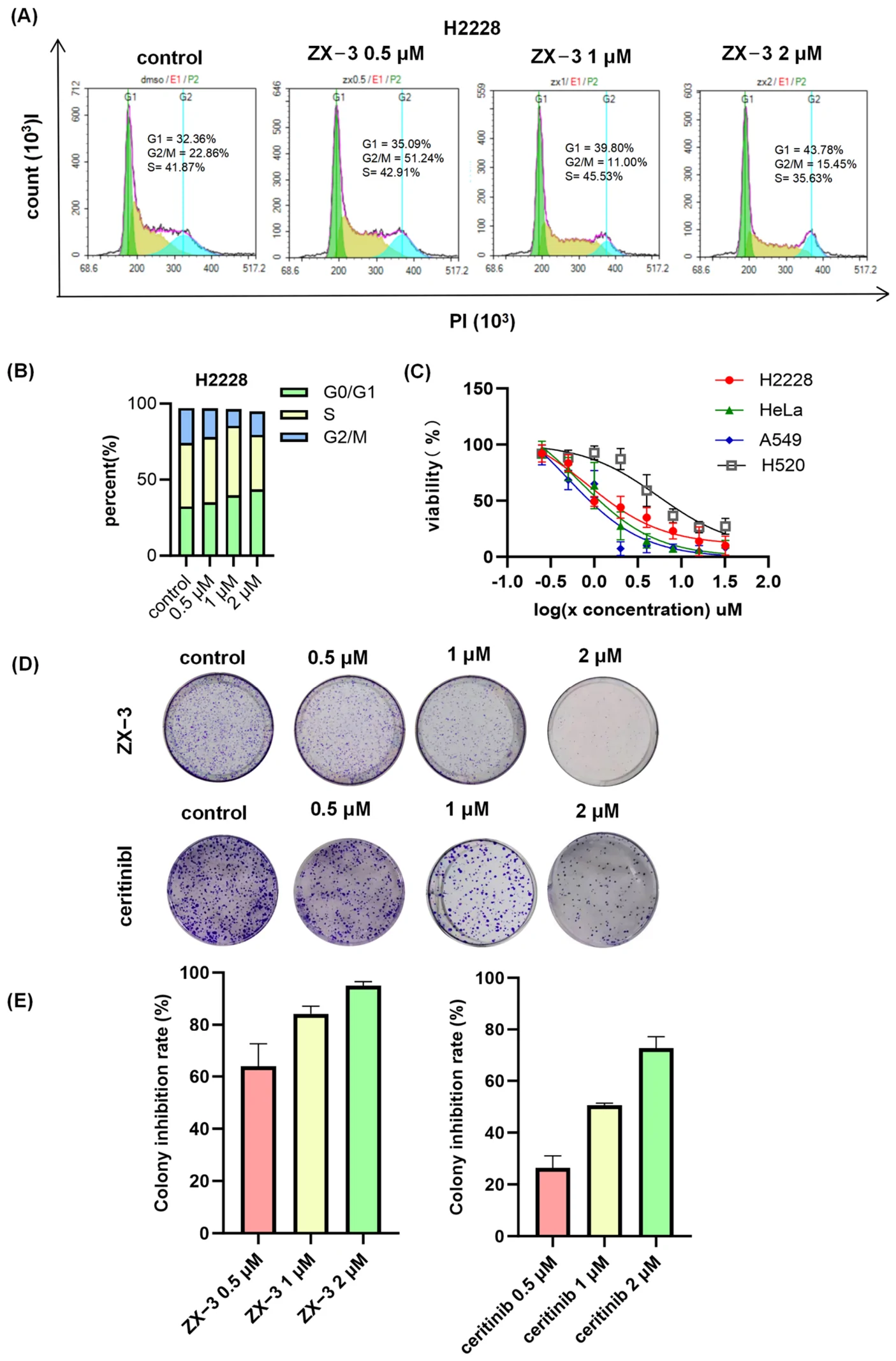
(**A**) ZX-3 cell arrested in G1 phase after 24 h treatment. (**B**) Quantitative analysis of (**A**). (**C**) The cytotoxicity of ZX-3 in high-EGFR-expressing and low-EGFR-expressing cells. (**D**) ZX-3 and ceritinib inhibit colony formation. (**E**) Quantitative analysis of (**D**).

**Figure 9:**
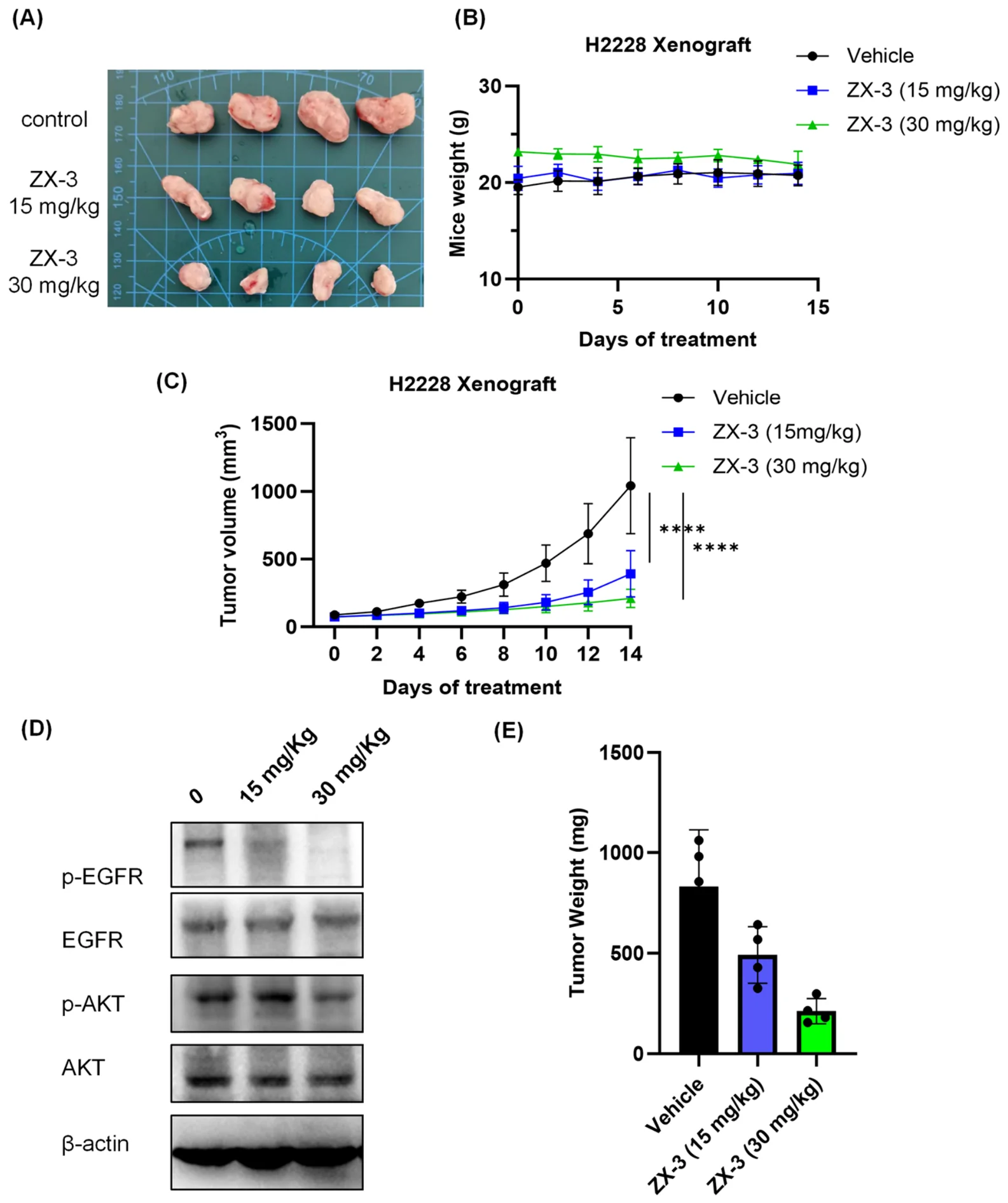
In vivo antitumor activity of ZX-3 in the H2228 xenograft model. (**A**) Tumor images of the treatment group and control group; (**B**) changes in body weight of mice in the treatment group versus the control group; (**C**) changes in tumor volume between the treatment group and the control group; (**D**) Western blot analysis results showing differences in EGFR and AKT protein phosphorylation levels in tumor tissues between the treatment group and the control group; (**E**) tumor weight. Data are expressed as mean ± standard error, *n* = 4. (****), *p* < 0.0001.

**Table 1:** In vitro cytotoxicity for all compounds in H2228 and MCF7 cell lines.

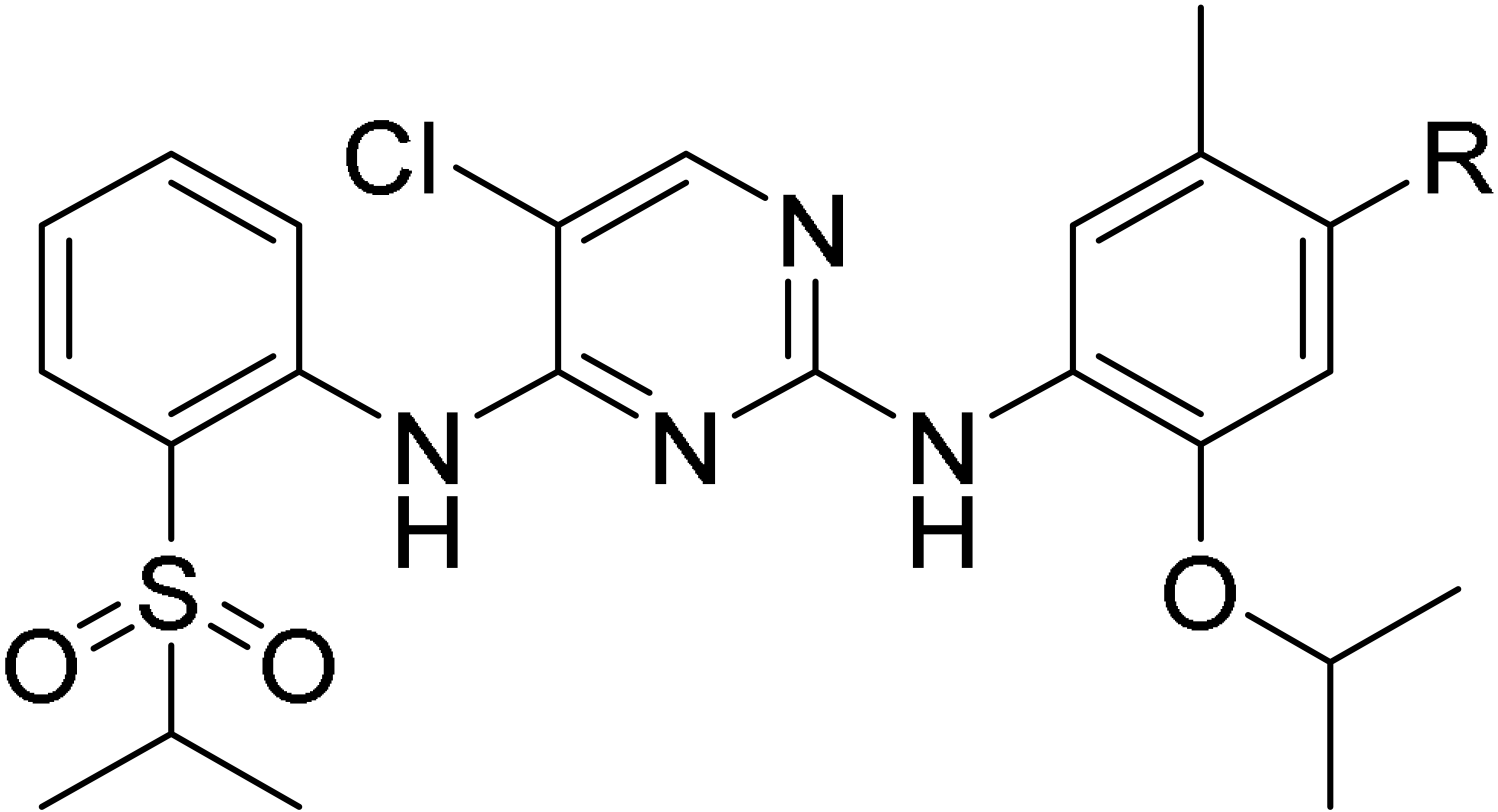			
Compound	R	IC_50_ (μM)	
		H2228	MCF7
ZX-3	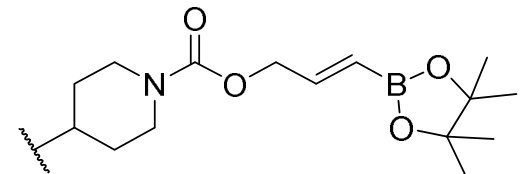	0.65 ± 0.11	1.26 ± 0.12
ZX-3a	-NHFmoc	>64	>64
ZX-3b	-NH_2_	6.25 ± 0.28	8.36 ± 0.51
ceritinib	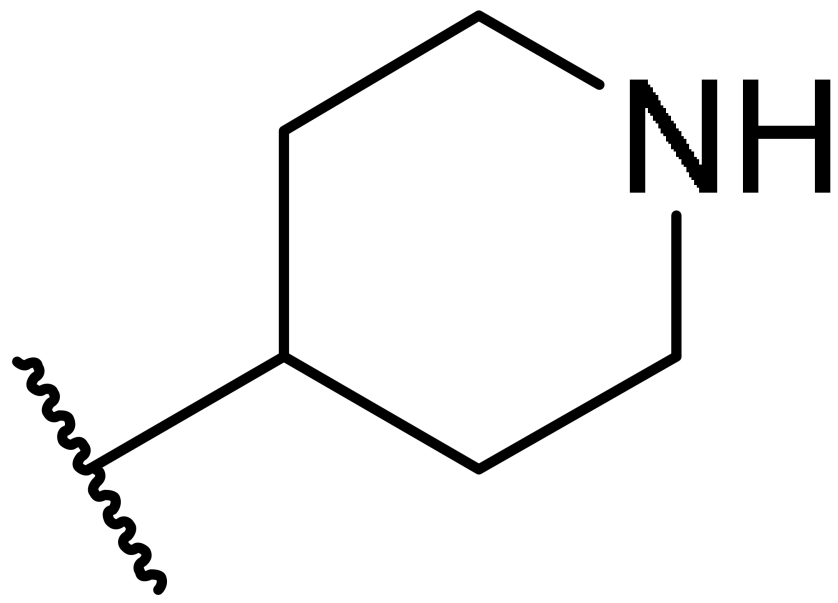	1.18 ± 0.14	4.33 ± 0.16
gefitinib	-	2.46 ± 0.33	2.65 ± 0.25

Data were obtained as the mean of three replicates.

**Table 2:** In vitro cytotoxicity for all compounds in LO2 cell line.

Compound	ZX-3	Ceritinib	Gefitinib
IC_50_ (μM)	4.28 ± 0.19	2.54 ± 0.21	20.35 ± 0.78
SI (IC_50_ (LO2)/IC_50_ (H2228))	6.58	2.15	7.68

Data were obtained as the mean of three replicates.

**Table 3:** EGFR kinase inhibition activity for all compounds.

Compound	ZX-3	ZX-3a	ZX-3b	Ceritinib	Gefitinib
IC_50_ (nM)	12.36 ± 0.69	>200	>200	>200	36.12 ± 1.35

Data were obtained as the mean of three replicates.

**Table 4:** The docking assay of ZX-3, ceritinib, and gefitinib with EGFR.

Compound	ZX-3	Ceritinib	Gefitinib
Docking score (kcal/mol)	−9.3	−7.2	−8.5

## Data Availability

The raw data supporting the conclusions of this article will be made available by the authors on request.

## Supplementary Materials

The following supporting information can be downloaded at https://www.mdpi.com/article/10.3390/molecules31193440/s1. Copies of ^1^H NMR, ^13^C NMR, and HRMS spectrograms are in the Supplementary Materials.

## References

[1] Bray F., Laversanne M., Sung H., Ferlay J., Siegel R.L., Soerjomataram I., Jemal A. (2024). Global cancer statistics 2022: GLOBOCAN estimates of incidence and mortality worldwide for 36 cancers in 185 countries. CA Cancer J. Clin..

[2] Hendriks L.E.L., Remon J., Faivre-Finn C., Garassino M.C., Heymach J.V., Kerr K.M., Tan D.S.W., Veronesi G., Reck M. (2024). Non-small-cell lung cancer. Nat. Rev. Dis. Prim..

[3] Liang X., Zhu X., Zhang Y., Chen M., Liu N. (2026). Targeting shared mechanisms of cisplatin resistance and metastasis in lung cancer for novel therapeutic strategies. Discov. Oncol..

[4] Zhou J., Kang Y., Chen L., Wang H., Liu J., Zeng S., Yu L. (2020). The Drug-Resistance Mechanisms of Five Platinum-Based Antitumor Agents. Front. Pharmacol..

[5] Passaro A., Jänne P.A., Mok T., Peters S. (2021). Overcoming therapy resistance in EGFR-mutant lung cancer. Nat. Cancer.

[6] Wang Y., Liu C., Chen H., Jiao X., Wang Y., Cao Y., Li J., Zhang X., Sun Y., Zhuo N. (2024). Clinical efficacy and identification of factors confer resistance to afatinib (tyrosine kinase inhibitor) in EGFR-overexpressing esophageal squamous cell carcinoma. Signal Transduct. Target Ther..

[7] Hirsch F.R., Scagliotti G.V., Mulshine J.L., Kwon R., Curran W.J., Wu Y.-L., Paz-Ares L. (2017). Lung cancer: Current therapies and new targeted treatments. Lancet.

[8] Liu Q., Yu S., Zhao W., Qin S., Chu Q., Wu K. (2018). EGFR-TKIs resistance via EGFR-independent signaling pathways. Mol. Cancer.

[9] Sigismund S., Avanzato D., Lanzetti L. (2017). Emerging functions of the EGFR in cancer. Mol. Oncol..

[10] Zhao J., Xu W., Zhou F., Zhang X., Zhou M., Miao D., Yu L., Zhang Y., Fan J., Zhou C. (2026). Navigating the landscape of EGFR TKI resistance in EGFR-mutant NSCLC—Mechanisms and evolving treatment approaches. Nat. Rev. Clin. Oncol..

[11] Lau S.C.M., Batra U., Mok T.S.K., Loong H.H. (2019). Dacomitinib in the Management of Advanced Non-Small-Cell Lung Cancer. Drugs.

[12] Wee P., Wang Z. (2017). Epidermal Growth Factor Receptor Cell Proliferation Signaling Pathways. Cancers.

[13] Wang Z., Li Y., He X., Fu Y., Li Y., Zhou X., Dong Z. (2023). In vivo evaluation of the pharmacokinetic interactions between almonertinib and rivaroxaban, almonertinib and apixaban. Front. Pharmacol..

[14] Deeks E.D. (2021). Furmonertinib: First Approval. Drugs.

[15] Hossam M., Lasheen D.S., Abouzid K.A. (2016). Covalent EGFR Inhibitors: Binding Mechanisms, Synthetic Approaches, and Clinical Profiles. Arch. Pharm..

[16] Chen L., Fu W., Feng C., Qu R., Tong L., Zheng L., Fang B., Qiu Y., Hu J., Cai Y. (2017). Structure-based design and synthesis of 2,4-diaminopyrimidines as EGFR L858R/T790M selective inhibitors for NSCLC. Eur. J. Med. Chem..

[17] Hu S., Tong L., Qin Q., Wen J., Li Y., Feng F., Wu K., Zhou Y., Shang J., Wang J. (2024). Design, Synthesis, and Biological Evaluation of Novel Diaminopyrimidine Macrocycles as Fourth Generation Reversible EGFR Inhibitors That Overcome Clinical Resistance to Osimertinib Mediated by C797S Mutation. J. Med. Chem..

[18] Marsilje T.H., Pei W., Chen B., Lu W., Uno T., Jin Y., Jiang T., Kim S., Li N., Warmuth M. (2013). Synthesis, Structure–Activity Relationships, and in Vivo Efficacy of the Novel Potent and Selective Anaplastic Lymphoma Kinase (ALK) Inhibitor 5-Chloro-N2-(2-isopropoxy-5-methyl-4-(piperidin-4-yl)phenyl)-N4-(2-(isopropylsulfonyl)phenyl)pyrimidine-2,4-diamine (LDK378) Currently in Phase 1 and Phase 2 Clinical Trials. J. Med. Chem..

[19] António J.P.M., Russo R., Carvalho C.P., Cal P.M.S.D., Gois P.M.P. (2019). Boronic acids as building blocks for the construction of therapeutically useful bioconjugates. Chem. Soc. Rev..

[20] Diaz D.B., Yudin A.K. (2017). The versatility of boron in biological target engagement. Nat. Chem..

[21] Dong S., Yousefi H., Savage I.V., Okpechi S.C., Wright M.K., Matossian M.D., Collins-Burow B.M., Burow M.E., Alahari S.K. (2022). Ceritinib is a novel triple negative breast cancer therapeutic agent. Mol. Cancer.

[22] Sampson J., Ju H.-M., Zhang N., Yeoh S., Choi J., Bayliss R. (2024). Targeting ERBB3 and AKT to overcome adaptive resistance in EML4-ALK-driven non-small cell lung cancer. Cell Death Dis..

[23] Voena C., Di Giacomo F., Panizza E., D’Amico L., E Boccalatte F., Pellegrino E., Todaro M., Recupero D., Tabbò F., Ambrogio C. (2013). The EGFR family members sustain the neoplastic phenotype of ALK+ lung adenocarcinoma via EGR1. Oncogenesis.

[24] Akçay G., A Belmonte M., Aquila B., Chuaqui C., Hird A.W., Lamb M.L., Rawlins P.B., Su N., Tentarelli S., Grimster N.P. (2016). Inhibition of Mcl-1 through covalent modification of a noncatalytic lysine side chain. Nat. Chem. Biol..

[25] Wang S., Luo D., Pu C., Ma X., Zhang H., Feng Z., Deng R., Yu S., Liu Y., Huang Q. (2023). Discovery of the GSH responsive “Y-PROTACs” targeting ALK and CDK4/6 as a potential treatment for cancer. Eur. J. Med. Chem..

[26] Helfrich B.A., Raben D., Varella-Garcia M., Gustafson D., Chan D.C., Bemis L., Coldren C., Barón A., Zeng C., Franklin W.A. (2006). Antitumor Activity of the Epidermal Growth Factor Receptor (EGFR) Tyrosine Kinase Inhibitor Gefitinib (ZD1839, Iressa) in Non–Small Cell Lung Cancer Cell Lines Correlates with Gene Copy Number and EGFR Mutations but not EGFR Protein Levels. Clin. Cancer Res. Clin. Cancer Res..

[27] Dhingra S., Shrestha P., Chowdhury A., Zhou Z., Jois S.D., Vicente M.d.G.H. (2023). The Synthesis of BODIPY-TKI Conjugates and Investigation of Their Ability to Target the Epidermal Growth Factor Receptor. Targets.

[28] Mohammadzadeh-Asl S., Aghanejad A., Yekta R., de la Guardia M., Dolatabadi J.E.N., Keshtkar A. (2020). Kinetic and thermodynamic insights into interaction of erlotinib with epidermal growth factor receptor: Surface plasmon resonance and molecular docking approaches. Int. J. Biol. Macromol..

[29] Zhou Y., Liu X., Xue J., Liu L., Liang T., Li W., Yang X., Hou X., Fang H. (2020). Discovery of Peptide Boronate Derivatives as Histone Deacetylase and Proteasome Dual Inhibitors for Overcoming Bortezomib Resistance of Multiple Myeloma. J. Med. Chem..

[30] Kang C.H., Kim E.-Y., Kim H.R., Lee C.O., Lee H.K., Jeong H.G., Choi S.U., Yun C.-S., Hwang J.Y., Lee J.-Y. (2016). Minor modifications to ceritinib enhance anti-tumor activity in EML4-ALK positive cancer. Cancer Lett..

[31] Yan G., Zhong X., Pu C., Yue L., Shan H., Lan S., Zhou M., Hou X., Yang J., Li D. (2021). Targeting Cysteine Located Outside the Active Site: An Effective Strategy for Covalent ALKi Design. J. Med. Chem..

[32] Wang A., Shuai W., Wu C., Pei J., Yang P., Wang X., Li S., Liu J., Wang Y., Wang G. (2024). Design, Synthesis, and Biological Evaluation of Dual Inhibitors of EGFRL858R/T790M/ACK1 to Overcome Osimertinib Resistance in Nonsmall Cell Lung Cancers. J. Med. Chem..

[33] Zheng L., Zhang Y., Mei S., Xie T., Zou Y., Wang Y., Jing H., Xu S., Dramou P., Xu Z. (2024). Discovery of a Potent Dual Son of Sevenless 1 (SOS1) and Epidermal Growth Factor Receptor (EGFR) Inhibitor for the Treatment of Prostate Cancer. J. Med. Chem..

