# Supplementary material for: Novel 2,4-Pyrimidinediamine Derivative as Potent EGFR Inhibitor for NSCLC Treatment
Source: Molecules. 2026 Sep 27;31(19):3440. doi: 10.3390/molecules31193440 (PMC13666482; doi:10.3390/molecules31193440)
Supplement: Supplementary file 1 [file molecules-31-03440-s001.zip › molecules-4533641-supplementary.pdf]

Supporting Information

Novel 2,4-pyrimidinediamine derivative as potent EGFR inhibitor for NSCLC treatment

Shiqi Jia, Xiaoling Lv, Jia Lv and Yiran Wu

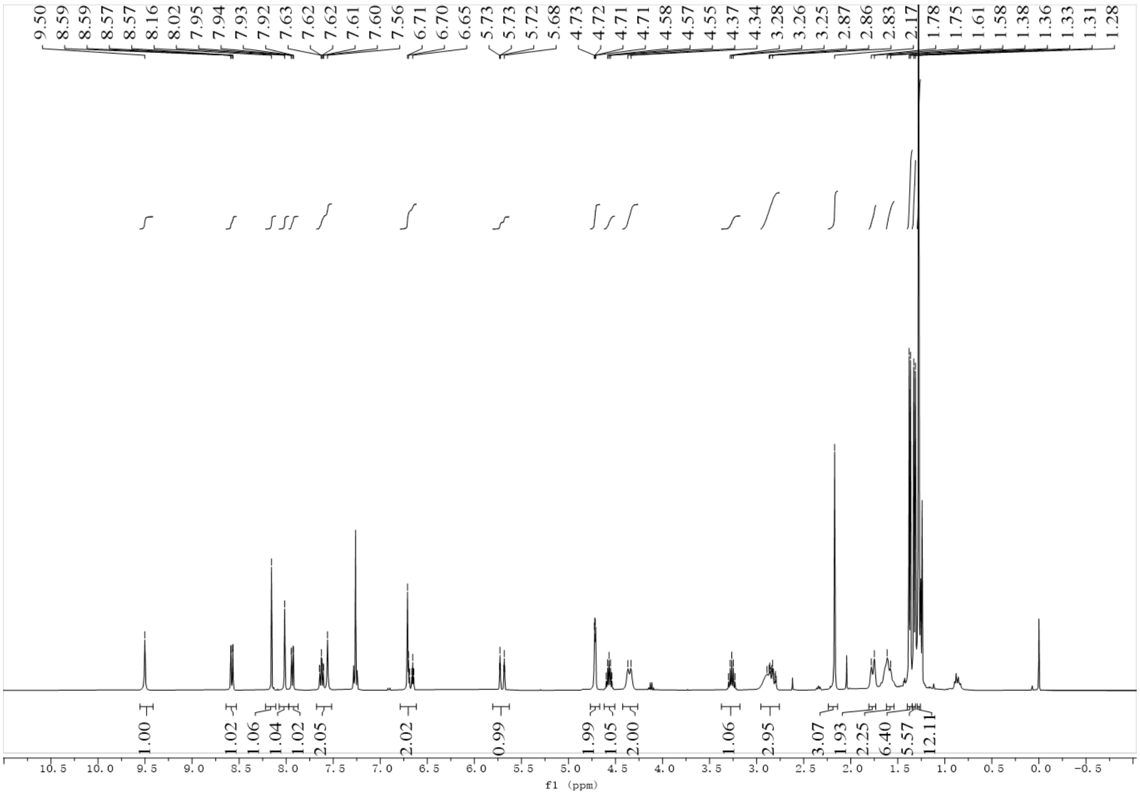

Figure S1: ^{1}H NMR spectrum of ZX-3

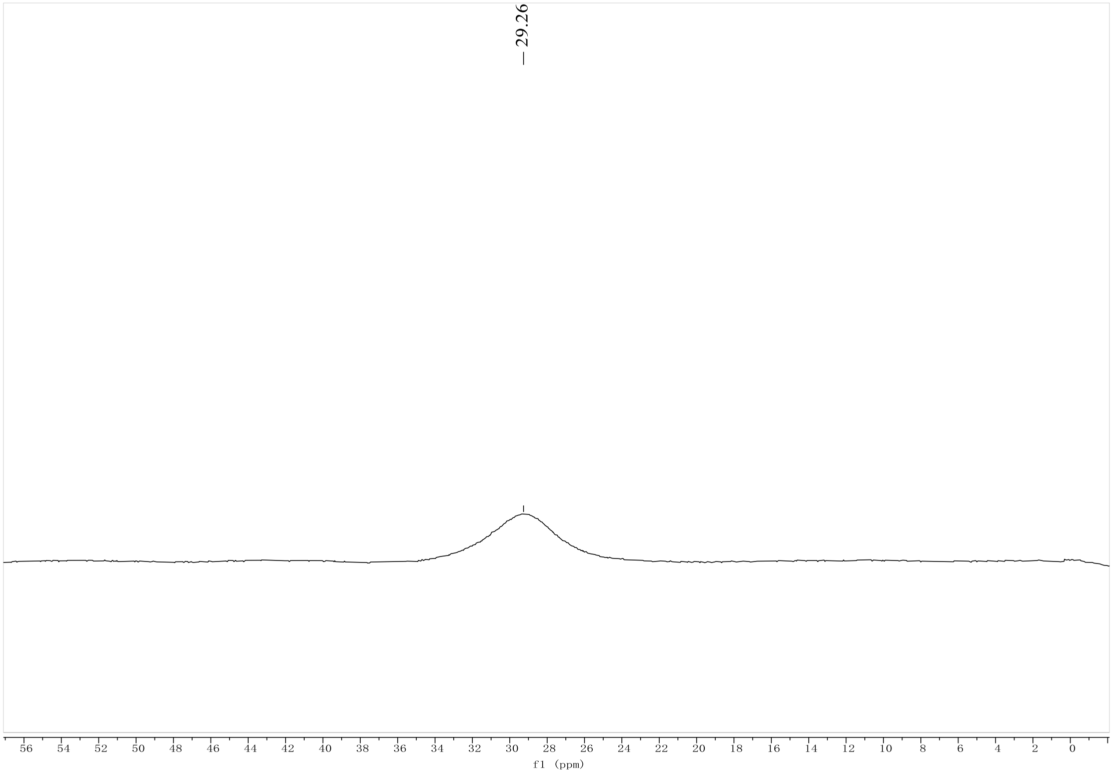

Figure S2: ^{11}B NMR spectrum of ZX-3

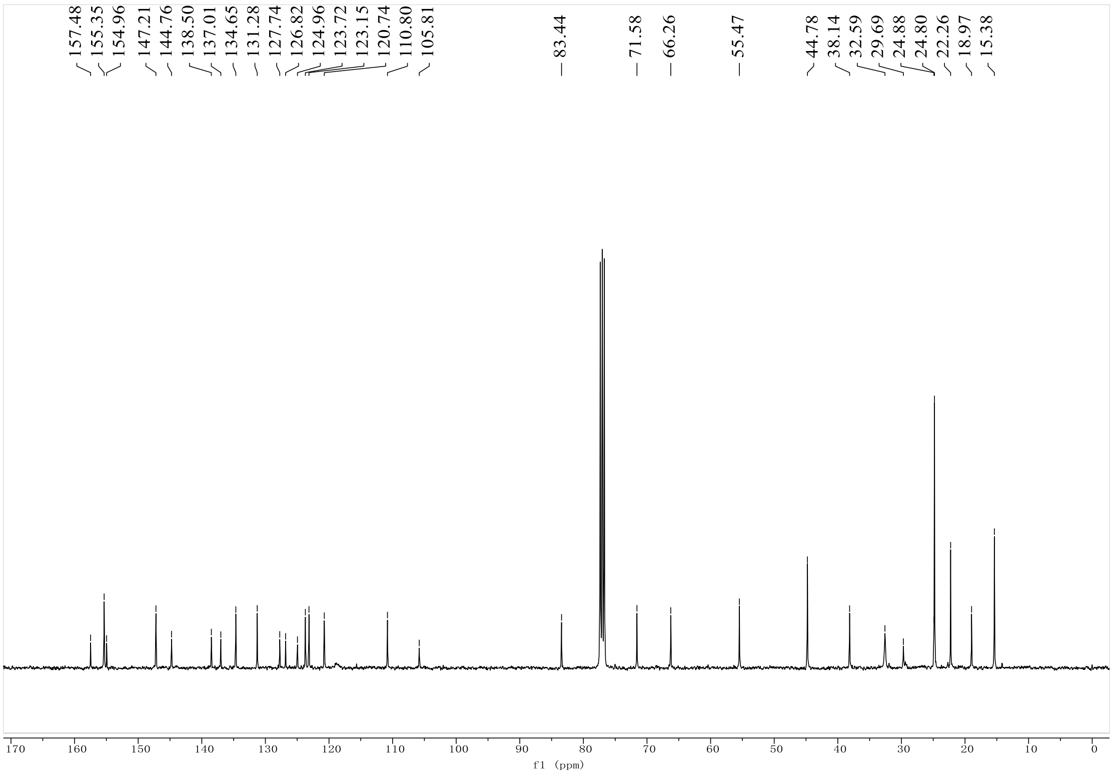

Figure S3: ^{13}C NMR spectrum of ZX-3

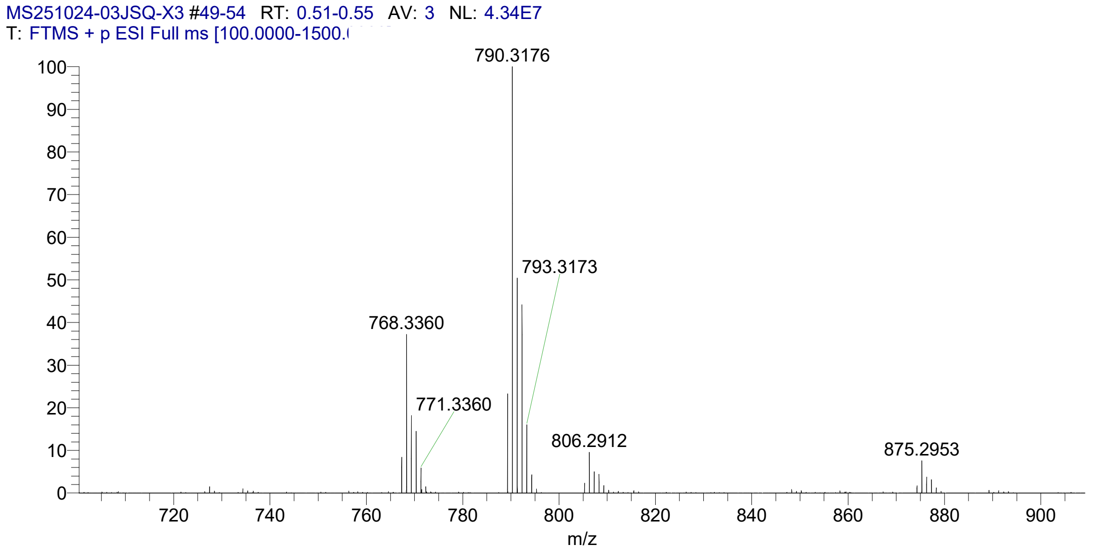

Figure S4: HRMS spectrum of ZX-3

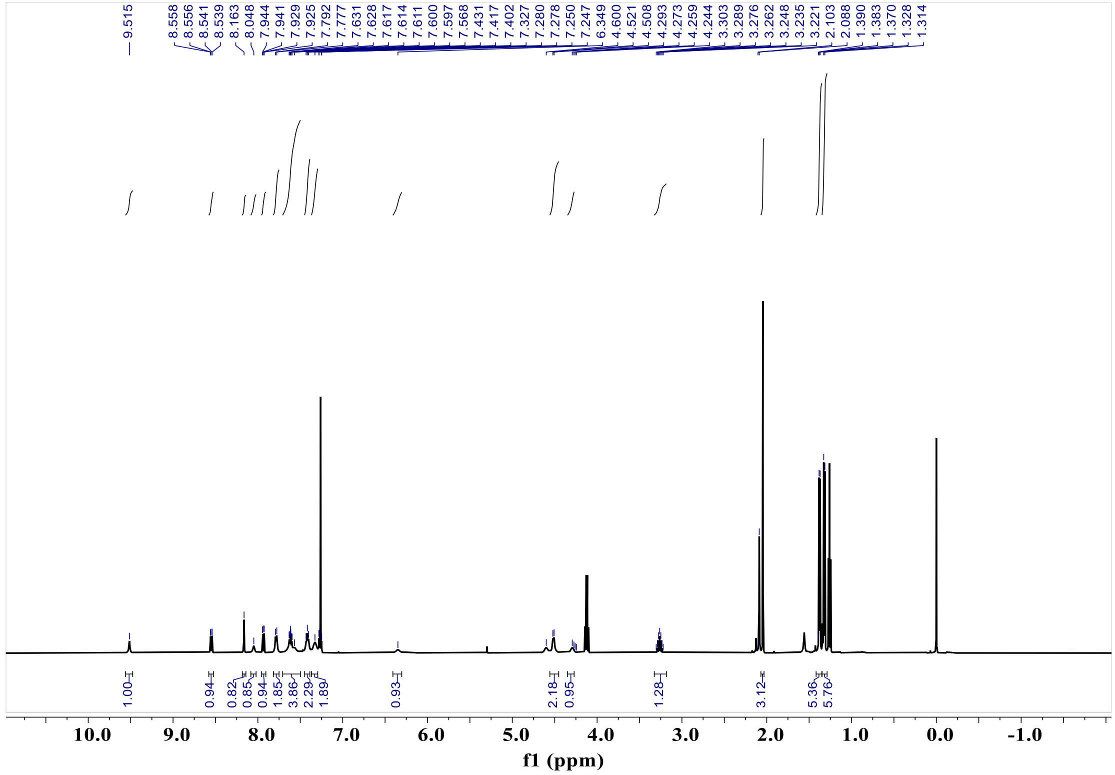

Figure S5: ^{1}H NMR spectrum of ZX-3a

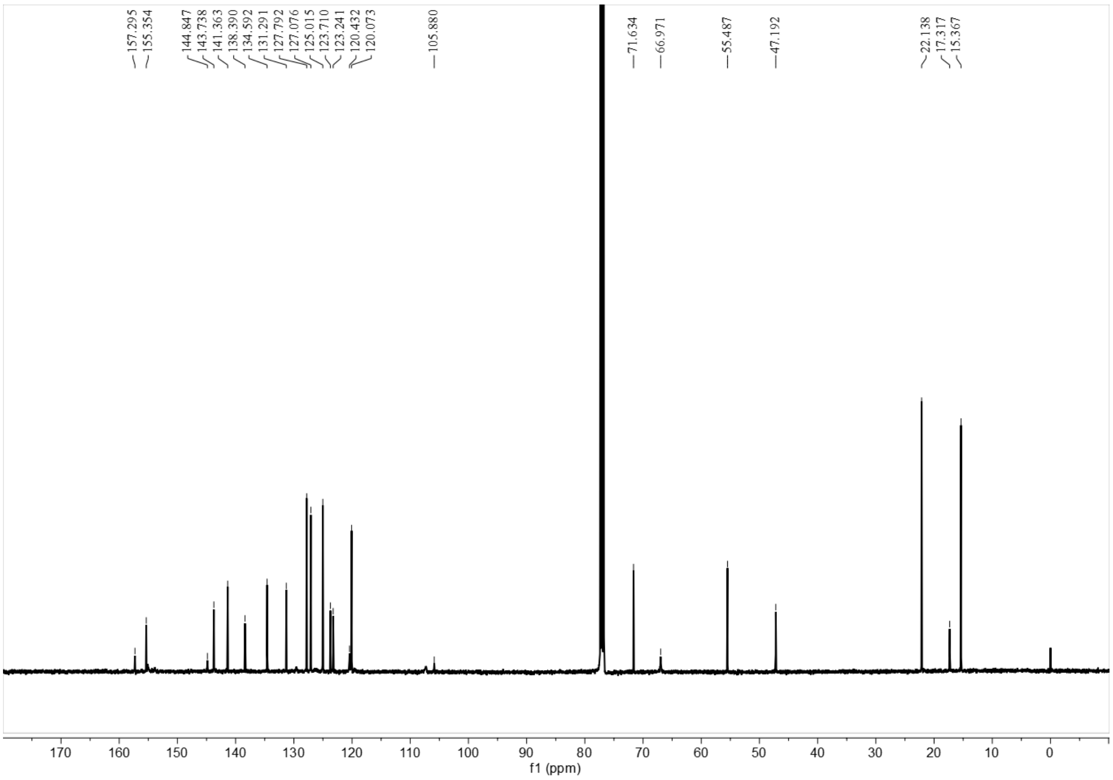

Figure S6: ^{13}C NMR spectrum of ZX-3a

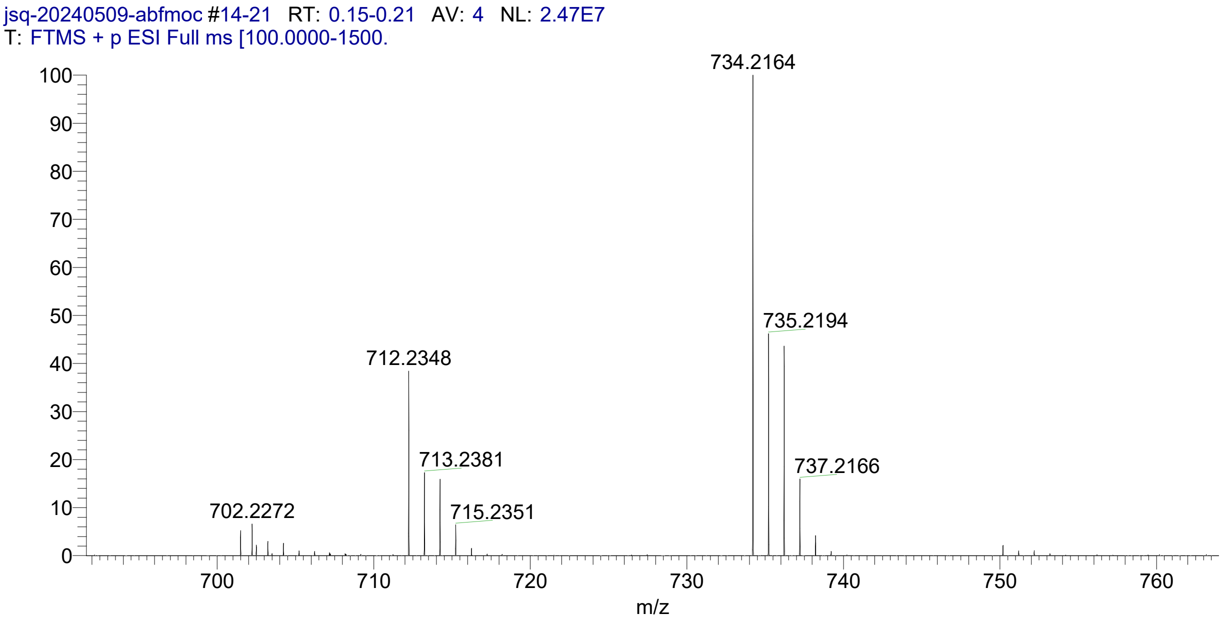

Figure S7: HRMS spectrum of ZX-3a

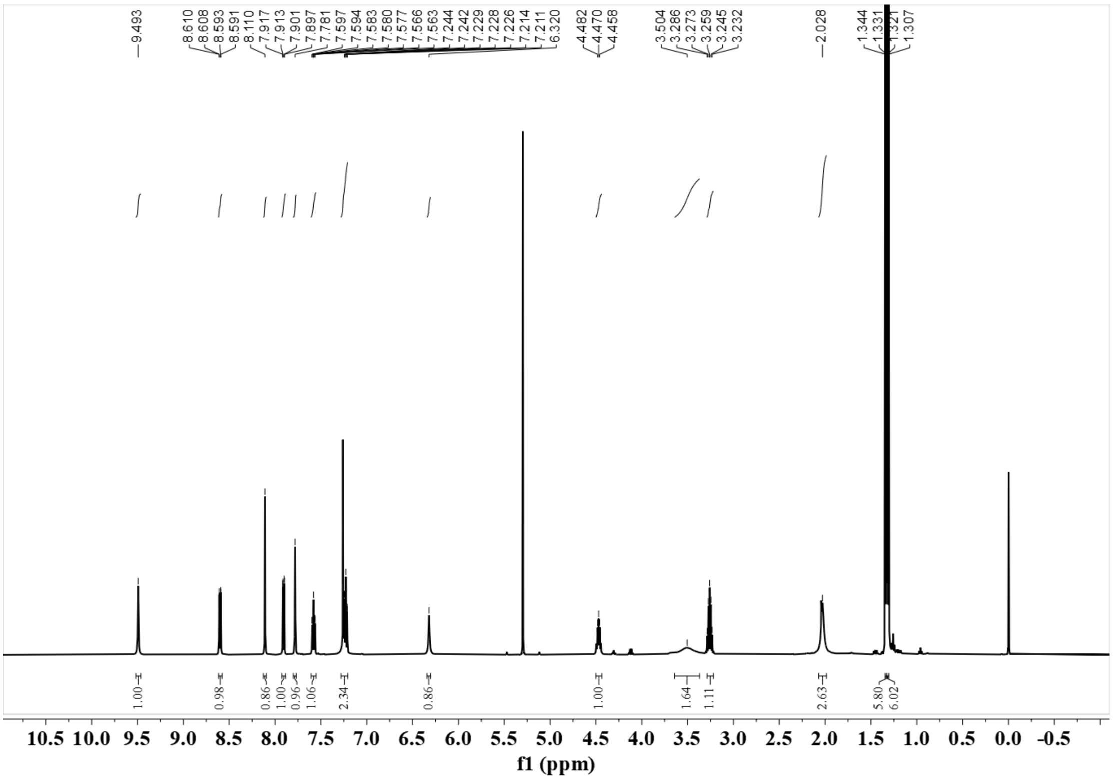

Figure S8: ^{1}H NMR spectrum of ZX-3b

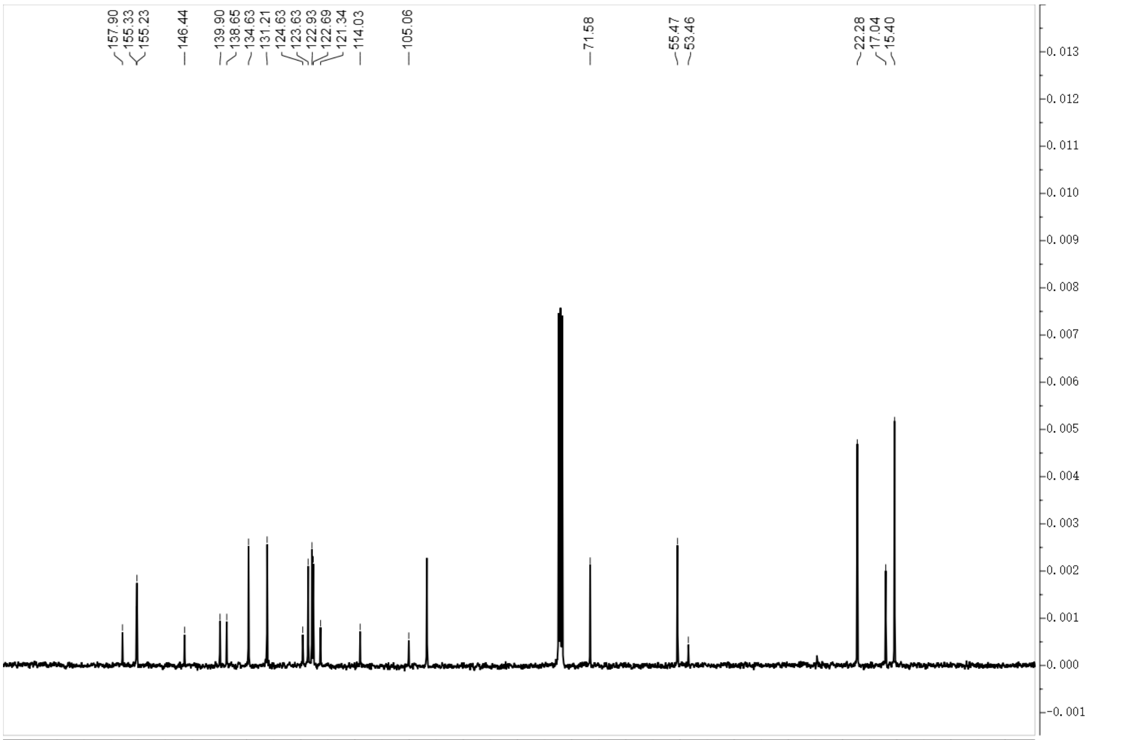

Figure S9: ^{13}C NMR spectrum of ZX-3b

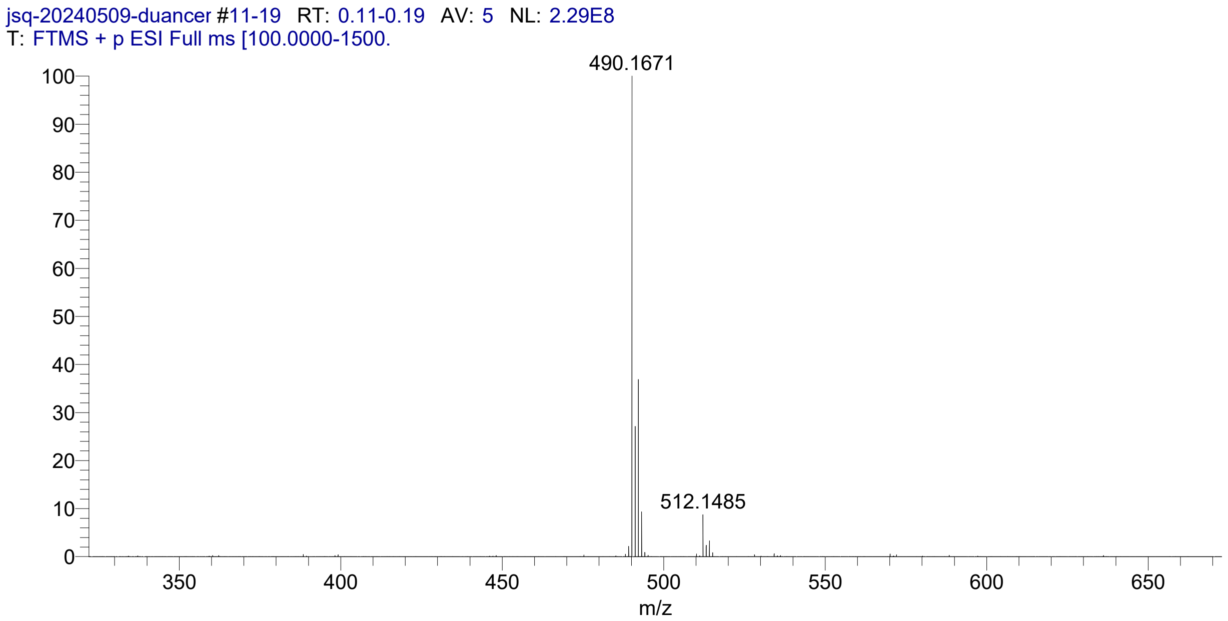

Figure S10: HRMS spectrum of ZX-3b
